# Supplementary material for: Understanding experiences of participating in a weight loss lifestyle intervention trial: a qualitative evaluation of South Asians at high risk of diabetes
Source: BMJ Open. 2014 Jun 20;4(6):e004736. doi: 10.1136/bmjopen-2013-004736 (PMC4067864; doi:10.1136/bmjopen-2013-004736)
Supplement: Author's manuscript [file bmjopen-2013-004736.draft_revisions.pdf]

Understanding experiences of participating in a weight loss lifestyle intervention trial: qualitative evaluation of South Asians at high risk of diabetes

Journal:	*BMJ Open*
Manuscript ID:	bmjopen-2013-004736
Article Type:	Research
Date Submitted by the Author:	20-Dec-2013

Complete List of Authors:	Morrison, Zoe; Centre for Population Health Sciences, The University of Edinburgh<br>Douglas, Anne; University of Edinburgh, Centre for Population Health Sciences<br>Bhopal, Raj; University of Edinburgh, Centre for Population Health Sciences<br>Sheikh, Aziz; University of Edinburgh, Division of Community Health Sciences; University of Edinburgh, Centre for Population Health Sciences

**Primary Subject Heading**:	Diabetes and endocrinology
Secondary Subject Heading:	Public health, Qualitative research, Research methods
Keywords:	DIABETES & ENDOCRINOLOGY, General diabetes < DIABETES & ENDOCRINOLOGY, PREVENTIVE MEDICINE, PUBLIC HEALTH, QUALITATIVE RESEARCH

SCHOLARONE™
Manuscripts

For peer review only

Understanding experiences of participating in a weight loss lifestyle intervention trial: qualitative evaluation of South Asians at high risk of diabetes

[1]Zoe Morrison PhD, [1]Anne Douglas MA, [1]Raj Bhopal DSE and [1,2]Aziz Sheikh FRCP on behalf of the trial investigators*

*Trial investigators: John F Forbes, Jason M R Gill, Michael Lean, John McKnight, Gordon Murray, Naveed Sattar, JaakkoTuomilehto and Sarah H Wild

[1]Centre for Population Health Sciences, The University of Edinburgh, Teviot Place, Edinburgh EH8 9AG, UK

[2]Division of General Internal Medicine and Primary Care, Brigham and Women's Hospital/Harvard Medical School, 3rd Floor, 1620 Tremont Street, Boston MA 02120-1613, USA

Corresponding author
Zoe Morrison,
Edinburgh Ethnicity and Health Research Group
Centre for Population Health Sciences,
Medical School, Teviot Place,
Edinburgh EH8 9AG, UK
Telephone: +44 131 651 4147
Fax: +44 131 650 6909
Email: zoe.morrison@ed.ac.uk

Keywords: diabetes mellitus; randomised controlled trial; South Asians; qualitative research, weight control

For peer review only

Abstract

Objective: To explore the reasons for enrolling, experiences of participating and reasons for remaining in a family-based, cluster randomised controlled trial of a dietitian-delivered lifestyle modification intervention aiming to reduce obesity in South Asians at high risk of developing diabetes.

Design: Qualitative study using narrative interviews of a purposive sample of trial participants following completion of the intervention. Data were thematically analysed.

Setting: The intervention was conducted in Scotland and resulted in a modest decrease in weight, but did not statistically reduce the incidence of diabetes.

Participants: We conducted 21 narrative interviews with 24 participants (20 trial participants and four family volunteers).

Results: Many participants were motivated to participate because of: known family history of diabetes and the desire to better understand diabetes related risks to their own and their family's health; ways to mitigate these risks; and to benefit from personalised monitoring. Home-based interventions, communication in the participant's chosen language(s) and continuity in dietitians supported their continuing engagement with the trial. Adaptations in food choices were initially accommodated by participants, although social and faith-based responsibilities were reported as important barriers to persevering with agreed dietary goals. Many participants reported that increasing their level of physical activity was difficult given their long working hours, physically demanding employment and domestic commitments; this being compounded by Scotland's challenging climate and a related reluctance to exercise in the outdoors.

Conclusions: Although participants had strong personal interests in participation and found the information provided by dietitians useful, they nonetheless struggled to incorporate the dietary and exercise recommendations into their daily lives. In particular, increasing levels of physical exercise was described as an additional and in some cases unachievable burden. Consideration needs to be given to strengthening and supporting lifestyle interventions with community-based approaches in order to help overcome wider social and environmental factors.

Article summary

Article focus

- Understanding experiences of participation and reasons for enrolling and remaining in a family-based, cluster randomised controlled trial of a dietitian-delivered lifestyle modification intervention aiming to reduce obesity in South Asians at high risk of developing diabetes.

Key messages

- Participants had strong personal interests in participation and found the information provided by dietitians useful but struggled to incorporate the dietary and exercise recommendations into their daily lives.
- Increasing levels of physical exercise was described as an additional and in some cases unachievable burden.
- We advocate consideration of community-based approaches to help overcome wider social and environmental factors when designing lifestyle interventions.

Strengths and limitations of this study

- We used family-based qualitative methods to understand the social and behavioural dimensions of a culturally adapted randomised lifestyle change trial in Scotland.
- A limitation of this study was the use of interpreters during data generation. We sought to give participants the opportunity to participate in a language of their choosing and could not achieve this without synchronous translation.

Introduction

People of South Asian origin are up to five times more likely than White Scottish adults to develop type II diabetes and, once established, are at particularly high risk of poor outcomes.[1-2] Dietary management and physical activity have been recommended as effective contributors to the prevention of type II diabetes mellitus.[3-7] We undertook an open, family-based, cluster randomised controlled trial that aimed to establish the benefits and cost-effectiveness of a complex dietitian-led dietary- and physical activity-based intervention for reducing obesity and preventing type II diabetes mellitus in people of Indian- and Pakistani-origin at high risk of developing diabetes living in Scotland. Participants were randomised to a tailored intervention, comprising 15 contacts with the trial dietitians over a three year period, or a low-intensity intervention, comprising four visits in the corresponding period.[8]

Cultural adaption of models of care for minority populations is known to be important in promoting equal access to care and self-efficacy[9] and requires inclusion of minority groups in healthcare research.[10] Ensuring participation and retention of these populations in longitudinal studies is a concern.[11,12] Barriers to effective engagement with minority ethnic communities include logistical considerations,[13] language-related barriers,[14] and issues of trust and respect between researchers and marginalised communities.[14,15] Extensive cultural adaptations were built into the trial intervention,[16] specifically flexible home-based interventions by the same dietitian, multilingual written resources and verbal communication, and modification of high-calorie traditional dishes.

The majority of recruitment was achieved informally through personal communications based upon trusted relationships such as faith-based activities and social groups including lunch clubs, referral by friends and general practitioners, community locations such as specialist food and clothing shops.[12] Participant retention rate of 97.7% far exceeded expectation. Outcomes of the trial were variable for individual participants. Overall the tailored intervention led to weight loss ≥2·5 Kg for 33 participants compared with 12 in the control group. The intervention group spent 3·0 hrs per week on physical activity compared to the control group's 2·1 hours, with little difference in food shopping and food preparation time. These results are reported in full separately.[17] We aimed to obtain a rich and multi-faceted understanding of reasons for trial participation and retention, and factors influencing adherence to the lifestyle intervention to give insight into the trial results.

Methods

We undertook a qualitative study[18] to inform our understanding of patient experiences of the trial processes.[19] We used narrative-based interviewing[20] in preference to interviews that utilise structured topic guides. Asking participants to tell their story allows people to spontaneously construct an account of their own experiences in a manner that is strongly shaped by and reflective of their cultural preferences. This method has been found to be an effective method of generating and interpreting experience-centred, culturally orientated data with people of South Asian backgrounds.[21]

The summary CONSORT flowchart is shown at Figure 1. Details of the trial participant population are shown in Table 1. We focused upon trial participants and family volunteers exiting the study. We utilised purposeful sampling[22] to ensure representation of the diversity within the trial population by sex, ethnicity, faith group, geographical location (Glasgow and Edinburgh), and whether they were allocated to the intervention or control group.[8]

During their final trial visit, participants were invited to take part in the qualitative study and given an information sheet. The trial dietitians then obtained consent to be contacted from those who were

For peer review only

willing to consider participation. A selection of participants were then contacted to arrange a research meeting, at the beginning of which the study was explained again before written consent to take part was obtained by the researcher. All interviews were conducted in the language of the participants' choice; interpreting services were used, if necessary. All fieldwork was undertaken with due regard to maintaining the best interests of participants and, in particular, assuring their confidentiality and anonymity

We used preliminary qualitative work (unpublished) to identify open questions to prompt participants' stories, when necessary, including:

- What were the motivations, perceptions and attitudes that led you to express an interest and then agree to participate in this trial?
- What factors have influenced your ability to engage with, and be faithful to, the study interventions?
- What factors have influenced participants' decisions to remain involved in the trial?
- More generally, what were the most memorable events and experiences that influenced decisions to accept the invitation for screening, agree to participate, adhere to and remain enrolled in the trial?

Thematic data analysis[20] was concurrently undertaken using NVivio9 software,[23] allowing emerging themes to inform on-going data collection using the constant comparison method.[24] We actively considered alternative explanation cases and allowed for the researcher's reflexive analysis of interpretations.[20] Regular discussions of the emerging findings among the trial qualitative subgroup and the active seeking of disconfirming data further ensured the trustworthiness of findings. Data collection ceased when saturation could reasonably be assumed.[25] Fully anonymised study results were only shared with trial dietitians once the trial was closed.

Results

We conducted 21 narrative interviews with 24 participants (20 trial participants and four family volunteers). We achieved high quality data from a diverse sample, enabling spontaneous account of participants' experiences in their chosen language(s) and narrative structure. Our process of recruitment and data generation is shown in Figure 2. Qualitative study participants are summarised by geographical location in Table 2, by intervention, ethnicity, language and faith in Table 3 and detailed in full in Table 4. Findings are reported in conjunction with illustrative data in relation to experiences of participation and retention within the trial, and adherence to the intervention. Our analytical framework is summarised in Box 1. We did not identify significant points of differentiation between the intervention and control group participants. We did identify a small number of gender-specific considerations.

Participation and retention in the trial

Perceived benefits of participation in the trial, the accommodation of participant choice of language and location, and trusting relationships between trial investigators and participants contributed to the trial's very high retention rate.

For peer review only - http://bmjopen.bmj.com/site/about/guidelines.xhtml

Perspectives on potential benefits of participation

Participants could not always fully recall how they were recruited into the trial. Two participants described themselves as having joined the trial because they were *'on the borderline'* of having diabetes (I.15,18) and three specifically noted their desire to lose weight (I.8,13,19).Some participants had been attracted by the provision of health related information (I.3,5,6,7,10,12). Increased awareness was particularly attractive in the light of family histories of diabetes mellitus (I.4,10,13,14,15,17,19,11).

> *"My parents had diabetes and my father-in-law also had, so I have always had fear about it. In fact I consulted libraries also to gain knowledge about it."* (I.11)

Other participants were in contrast more ambivalent about the trial, participating on the basis of *'no harm'* rather than in pursuit of direct benefit (I.1,6,9).

Participant choice

The emphasis upon patient choice built into the trial design contributed to participant engagement and retention. Accommodating preferences for consultation at home or in clinic was appreciated as some welcomed the opportunity to get out (I.10,15) whilst others welcomed home consultations (I.4,9,17). However, not every participant kept a diary and home visits presented some scheduling difficulties that required out-of-hours working by dietitians, as one participant observed:

> *"You can't do it all the time because the people are at home at different times of the day, some are in the evening, some late evening, they are not there during the day time."* (I.9)

Being able to speak to female dietitians in their language(s) of choice was valued by female participants (I.4,7,11,13), with participants feeling free to take the approach best suited to them:

> *"It made me – like – relaxed when she was around because I didn't, sometimes I don't understand in English, then I could speak to her in Indian. But mostly I didn't want to speak to her in Indian."* (I.16)

Such preferences were less frequently expressed by men (I.18,19).

Relationships

Some participants decided to participate in the trial as a result of knowing members of the research team. In addition, the relationships that developed between participants and their dietitians over the course of the trial positively contributed to retention. Whilst participants did not commonly refer to or produce their written materials, they placed importance on good verbal communication (I.11,16), empathy and compassion (I.7,10,18).

> *"She used to advise me but so compassionately and afterwards I used to think over it that it is for my help ultimately I will be the one who will benefit she is not my relative still she cares so much."* (I.19)

Adherence to the intervention

Adherence to the trial proved a complex issue to investigate as participants were well-informed and reported the correct behaviours even if they were not pursuing these. Adherence involved different understandings of the advocated lifestyle changes and unanticipated motivations such as caring for other family members or a sense of obligation to the dietitian. Non-adherence to the trial was in some case culturally motivated, or due to contextual factors such as climate or lack of time.

Understanding adherence

Participants who felt they had adhered to the trial mostly described changes they had made in relation to frequency of exercise (I.11.12) and more informed food choices (I.4,10,11,17,18,21) Some felt that this had not been a particularly significant change as they had a balanced diet already (I.9,12). Conversely, some were evangelical about the trial and their related achievements. Some had lost significant amounts of weight (e.g. *"half a stone"* (I.11)). Two gentleman described how their lives had fundamentally changed, one having given up high alcohol consumption (I.15), the other having become a runner for charity:

> *"I could have never imagined …I could not even walk 2 kilometres then I have started doing 5 [km] then 10 [km] then I have done lot of 10 then I thought lets go for the bigger [half marathon]then it took me 2hours and 19 minutes …So with that time we raised £2,000 - I was in the paper!"* (I.19)

Many participants had difficulty in sustaining lifestyle changes (I.10,16,13,14) describing a process of *"fits and starts"* (I.10) as they struggled consistently to follow the advice given despite known health risks. This was true even subsequent to a diagnosis of diabetes (I.7,16). In contrast, looking after other family members influenced adherence. One participant attributed a weight loss of 8kg in part to different methods of food preparation (e.g. less frying) and their having increased their daily exercise due to the need to look after their son's dog (I.8). Another described how they had followed the intervention because they felt it would be beneficial to the whole family (I.11).

Scheduled consultations themselves impacted on levels of adherence as participants either avoided their appointments as they were on the *"downward slope"* (I.10,11) or adapted their behaviour in anticipation:

> *"She used to come in every three months so before she used to come I used to feel a kind of pressure to make sure that my weight had not increased."* (I.18)

Reasons for non-adherence

Culture considerations were evident in discussions regarding the challenges of adherence to the lifestyle intervention. The importance of food within South Asian cultures was referred to for example in the context of sharing home-made sweets with grandchildren (I.2,14) and traditional food preparation techniques:

> *"Once a week they have children all come so we feel that the food should be much nicer according to the tradition and also children don't like ordinary vegetables they fancy food like from McDonald's so just to compete with that kind of food we try to make our old Indo-Pakistani dishes."* (I.20)

The role of food in community functions (I.15) and faith were also given as challenges to adherence, with one participant feeling she couldn't avoid gaining weight

> *"I know that if I lose more weight I'm going to put it back on at Ramadan because I just have to walk by food and it just adds onto my body, the fat just is invisible and it runs after you."* (I.13)

Whilst the trial was designed to be culturally sensitive to the needs of South Asian populations, some members of the population felt that the advice did not accommodate their more international food preferences (I.10,13,15).

Other reasons for non-adherence related to wider contextual factors. Participants felt they were too busy to find the time to exercise (I.1,4,14,15,16). Two male participants noted the physical demands of their employment as a barrier to exercise (I.14,17), whilst some females felt unable to get enough time out of the house (I.2):

> *"I try to go for walks but it's hard to find time as I have so many grand-children and women in our community don't get out of the house that much. There is no one else who will come with me."* (I.4)

The climate was a consideration for South Asians living in Scotland as participants found recommended exercise such as walking problematic in the cold (I.1,2,15,16) and food was described as a cultural representation of warmth (I.2). It therefore seemed counter-intuitive to increase one without the other.

Discussion

We used qualitative methods to understand the social and behavioural dimensions of the first family-based, culturally adapted randomised lifestyle change trial to investigate approaches to reducing obesity and prevent type II diabetes mellitus in people of Indian- and Pakistani-origin living in Scotland. Findings indicate that individuals may not necessarily prioritise their own health over other factors when making lifestyle choices despite known risks and prevention strategies. Participants were attracted to the trial by the availability of regular health monitoring and personalised information, particularly those who were aware of a family history of diabetes. The choice of home- or clinic-based interventions, communication in the participants' chosen language(s) and trusting relationships between participants and dietitians contributed to the high retention rate. Participants stated that they did not find adherence to advice on food choices and preparation problematic, although some conceded that they did not always chose to take the advice, citing examples of community and faith based considerations that made consistent adherence difficult. Participants did find increasing their levels of physical activity problematic given demands such as the Scottish climate, long working hours, physically demanding jobs and domestic commitments.

One important limitation of this study was the use of interpreters during data generation. We sought to give participants the opportunity to participate in a language of their choosing and could not achieve this without synchronous translation. During the initial stages of the study retrospective quality checking of interview audio and transcripts highlighted that the interpreter was not accurately reporting the views of the participants. We addressed this by recruiting a new interpreter and providing training as to the exact nature and purpose of the services required. A further limitation is the lack of inclusion of Indian Hindu participants and the small sample of family volunteers. For practical reasons (such as interview cancellation by participants) we could not achieve this within our sample during the time available.

In discussing our findings in the light of current literature on participation and retention we consider the benefits sought from the intervention, flexibility within the trial design and participant/dietitian relationships. Regarding adherence our participants talked about lifestyle changes and we have reported findings accordingly. In contrast, the literature considers attitudes to food and physical activity separately and we discuss our findings in this context.

The need for individualised and context specific information for people with pre-diabetes has been acknowledged[26] and was a benefit participants sought from engaging with the trial. People recognised they were at risk due to family histories. Losing weight was not articulated as a dominant sought-after

benefit even though it was a secondary outcome of the trial and this may be due to culturally situated traditional beliefs about body size. In South Asian communities a tendency to equate large size with healthy womanhood, marriage and reproduction, securing a good job and high social status has been identified,[27] although this view may be less prevalent now. Understanding of the causal relationship between weight and diabetes was less evident, re-iterating calls for education within South Asian communities on the causes and prevention of diabetes.[28]

Findings highlight the importance of accommodating participant choice in the design of complex lifestyle interventions. Appreciation of flexibility within diabetes prevention programmes has been recognised,[29] as has the need to overcome known barriers to effectively engaging with minority ethnic communities.[13-15] Whilst culturally sensitive adaptations are essential and were achieved within this trial[16] they should avoid reinforcing stereotypes,[52] for example by focusing on traditional dishes. This did not necessarily sufficiently accommodate complex patterns of dietary acculturation.[31]

Despite cultural adaptation, the trial written resources were rarely referred to. In contrast, verbal communication and participant/dietitian relationships were repeatedly referred to. The importance of non-judgemental professional attitudes, and empathy upon within self-management interventions has been recognised[26,29] and, together with known issues of trust and respect between researchers and marginalised communities, [14,15] was anticipated in the trial design. The intimate nature of this home based lifestyle intervention required a compassionate approach to investigation, out-of-hours working and an emphasis on participant choice different to that of the majority of interventions delivered in clinical settings. Dietitian skill in building relationships was felt to be very important in addition to skills in the verbal communication of advice and information. The importance of continuity in dietician staff was emphasised in trial design, but the strength of participant-dietitian relationships was not anticipated and proved to be significant in retention across the trial (99% in intervention group and 97% in control group). In some cases it seemed that participant loyalty was to the dietitian rather than the trial.

Our findings suggest that the cultural construction of food made it difficult for South Asians to make the healthy food choices advised despite known health risks. For members of this community, food is the focus of good living and etiquette.[27] In communities that have previously faced economic insecurity, being able to afford food is a sign of success, demonstrating social power and hospitality.[27,31,32]Although most migrants alter their eating habits following migration,[31] South Asian systemic beliefs regarding the heating and cooling properties and merits of food endure.[33] The role of food in community- and faith-based activities together with complex intergenerational patterns of dietary acculturation detracted from consistent adherence to both the tailored and low-intensity interventions.

It is known that the South Asian population has lower levels of physical activity than the general population and lesser intrinsic motivations for physical exercise.[34,35] We have corroborated proposed reasons for this, including lack of time and/or childcare[34,35] and gendered barriers to exercise, such as the availability of single-sex facilities and the depiction of exercise as a selfish act detracting from family care.[35] We further note the role of climate and the interplay between South Asian systemic beliefs regarding the heating and cooling properties and merits of food[33]and the demands of living and undertaking physical activity in the cold Scottish climate.

One insight from these findings is that well-informed individuals may not necessarily chose or feel able to act on health-related advice due to other lifestyle considerations. People sought health related information as the main benefit of participation and the dietary requirements of the trial were not felt

For peer review only

by participants to be an extra burden in their everyday life. However, culturally situated influences on food choices often took precedence over the advice and information given in both intervention and control groups despite acknowledged health implications.

The increased levels of physical activity were however seen to pose an additional burden by participants, again due to cultural factors including those previously identified, such as lower intrinsic motivation, a lack of time and/or access to appropriate facilities, and domestic considerations, each and all of which may impact upon the prevention and management of diabetes In addition, findings highlighted the role of climate in decisions relating to food and physical activity. This suggests that diet and exercise are less distinct within South Asian communities and indicates a need to better understand the impact of climate on lifestyle choices for immigrant populations.

Acknowledgements: We have previously thanked and named many individuals who contributed to the trial.(8) We further acknowledge the Trial Dietitians (Alyson Hutchison, Anu Sharma, Sunita Wallia and Ruby Bhopal), Nandini Choudhuri, Sujama Roy and Salman Aziz; current Trial Steering Committee (Prof Nigel Unwin (ex-chair), Prof Graham Hitman (chair), Dr Nita Forouhi, Dr Deepak Bhatnagar, Dr Ghada Zoubiane , and Mr Iqbal Anwar); the trial Data Monitoring and Ethics Committee (Prof Iain Crombie, Dr Mike Small, Dr Mike Kelly, Prof Kamlesh Khunti); South Asian Community & religious organisations and individuals who contributed to the recruitment efforts; and finally to all the participants who gave their time to take part in this study.

Funding: This study was funded by the National Prevention Research Initiative (G0501310), a funding consortium comprising the British Heart Foundation; Cancer Research UK; Department of Health; Diabetes UK; Economic and Social Research Council; Medical Research Council; Health & Social Care Research & Development Office for Northern Ireland; Chief Scientist Office, Scottish Government Health Directorate; the Welsh Assembly Government; and World Cancer Research Fund. Additional financial support was provided from NHS Lothian and NHS Greater Glasgow & Clyde R&D, Chief Scientist Office, NHS Health Scotland and NHS National Services Scotland.

Contributors: Zoe Morrison and Aziz Sheikh were responsible for the study design, supervised the data collection, and drafted the manuscript. Anne Douglas and Raj Bhopal designed the protocol for the larger study and assisted with obtaining consent for data collection together with other members of the trial team. Each of the authors was involved in data analysis and interpretation, critical review and refinement of the manuscript.

Competing interests: We declare that we have no conflict of interest.

Data sharing statement: Interview transcripts are available form the corresponding author at the University of Edinburgh, who will provide a permanent, citable and open access home for the dataset.

References

1. Fischbacher C. M., Bhopal R., Steiner M., Morris A. D., Chalmers J. Is there equity of service delivery and intermediate outcomes in South Asians with type 2 diabetes? Analysis of DARTS database and summary of UK publications. *Journal of Public Health*, 2009; 31: 2; 239 – 249. doi 10.1093/pubmed/fdp003.
2. Worth A, Irshad T, Bhopal R, Brown D, Lawton J, Grant E, Murray S, Kendall M, Adam J, Gardee R, Sheikh A. Vulnerability and access to care for South Asian Sikh and Muslim patients with life limiting illness in Scotland: prospective longitudinal qualitative study. *British Medical Journal,* 2009; 338: b183. doi: 10.1136/bmj.b183.
3. Li G, Zhang P, Wang J, Gregg EW, Yang W, Gong Q, et al. The long-term effect of lifestyle interventions to prevent diabetes in the China Da Qing Diabetes Prevention Study: a 20-year follow-up study. *Lancet* 2008 May 24;371(9626):1783-9.
4. Tuomilehto J, Lindstrom J, Eriksson JG, Valle TT, Hamalainen H, Ilanne-Parikka P, et al. Prevention of type 2 diabetes mellitus by changes in lifestyle among subjects with impaired glucose tolerance. *N Engl J Med* 2001 May 3;344(18):1343-50.
5. Knowler WC, Barrett-Connor E, Fowler SE, Hamman RF, Lachin JM, Walker EA, et al. Reduction in the incidence of type 2 diabetes with lifestyle intervention or metformin. *New England Journal of Medicine* 2002 Feb 7;346(6):393-403.
6. Gillies CL, Abrams KR, Lambert PC, Cooper NJ, Sutton AJ, Hsu RT, et al. Pharmacological and lifestyle interventions to prevent or delay type 2 diabetes in people with impaired glucose tolerance: systematic review and meta-analysis. *BMJ* 2007 Feb 10;334(7588):299.
7. Hopper I, Billah B, Skiba M, Krum H. Prevention of diabetes and reduction in major cardiovascular events in studies of subjects with prediabetes: meta-analysis of randomised controlled clinical trials. *Eur J Cardiovasc Prev Rehabil* 2011 Dec;18(6):813-23.
8. Douglas A, Bhopal RS, Bhopal R, Forbes JF, Gill JM, McKnight J, Murray G, Sattar N, Sharma A, Wallia S, Wild S, Sheikh A. Design and baseline characteristics of the PODOSA (Prevention of Diabetes & Obesity in South Asians) trial: a cluster, randomised lifestyle intervention in Indian and Pakistani adults with impaired glycaemia at high risk of developing type 2 diabetes. *BMJ Open*, 2013; Feb 22;3(2). pii: e002226. doi: 10.1136/bmjopen-2012-002226.
9. Griffiths, C, Motlib, J, Azad,A, Ramsay, J, Eldridge, S, Feder,G, Khanam, R, Munni, R, Garrett, M. Randomised controlled trial of a lay-led self-management programme for Bangladeshi patients with chronic disease. *Br J Gen Pract.* 2005 November 1; 55(520): 831–837.
10. Mason S, Hussain-Gambles M, Leese B, Atkin K, Brown J. Representation of South Asian people in randomised clinical trials: analysis of trials' data. *BMJ* 2003; 326: 1244–1245.
11. Sheikh A, Netuveli G, Kai J, Panesar SS. Comparison of reporting of ethnicity in US and European randomised controlled trials. *BMJ* 2004; 329: 87-8.
12. Samsudeen B S, Douglas A, Bhopal R. Challenges in recruiting South Asians into prevention trials; health professional and community recruiters' perceptions on the PODOSA trial. *Public Health* 2011; 125:201-209.
13. Hussain-Gambles M. South Asian patients' views and experiences of clinical trial participation. *Family Practice* 2004; 21: 636-42.
14. Stirland L, Halani L, Bhopal, R, et al. Recruitment of South Asians into asthma research: qualitative study of UK and US researchers. *Primary Care Respir J.* 2011 Sep;20(3):282-90. Doi:10.4104/pcrj.2011.00032
15. Harris, Y, Gorelick PB, Samuels P, Bempong, I. Why African Americans may not be participating in clinical trials. *J Natl Med Assoc* 1996: 88: 630-634.

For peer review only

16. Wallia S, Bhopal R.S., Douglas A, Bhopal R, Sharma A, Hutchison A, et al. Culturally adapting the prevention of diabetes and obesity in South Asians (PODOSA) trial. *Health Promot Int* 2013
17. Bhopal RS, Douglas A, Walia S, Fobes JF, Lean M, Gill JMR, et al. Weight change over three-years in South Asians at high risk of diabetes: effects and costs in a cluster-randomised trial of a lifestyle intervention. *Lancet Diab Endocrin* (in press)
18. Malterud K. Qualitative Research: standards, challenges and guidelines. *Lancet*, 2001:358; 483-488.
19. The PLOS Medicine Editors. Qualitative research: understanding patients' needs and experiences. *PLoS Med* 4(8): e258.doi:10.1371/journal.pmed.0040258
20. Riessman C K. *Narrative Methods for the Human Sciences,* London: Sage; 2008.
21. Squire C. Experience-centred and culturally orientated approaches to narrative. In: Andrews M, Quire C, and Tamboukou M. (eds.) *Doing Narrative Research,* London: Sage; 2008.
22. Patton MQ. (1990) *Qualitative evaluation and research methods.* 2nd ed. London: Sage Publications.
23. QSR International Pty Ltd. NVivo qualitative data analysis software Version 9. 2008.
24. Barbour, R. *Introducing Qualitative Research.* Sage: London; 2008.
25. Morse J. The significance of saturation. *Qualitative Health Research.* 1995;5(2):147-
26. Maindal et al. Lifting the lid of the "black intervention box" –the systematic development of an action competence programme for people with screen-detected dysglycaemia. *BMC Health Services Research* 2010,10: 114.
27. Bush, HM, Williams, RGA, Lean, MEJ and Anderson, AS. Body image and weight consciousness among South Asian, Italian and general population women in Britain. *Appetite*, 2001, 37:207-215.
28. Rankin, R and Bhopal, R. Understanding of heart disease and diabetes in a South Asian community: cross-sectional study tsting the 'snowball' sample method. *Public Health*, 2001; 253-260.
29. Walker, C, Hernan, A, Reddy, P and Dunbar, JA. "Sustaining modified behaviours learnt in a diabetes prevention program in regional Australia: the role of social context. *BMC Health Services Research* 2012, 12: 460.
30. Liamputtong, P. *Researching the vulnerable.* Sage: London; 2007.
31. Azar, KMJ, Chen, E. Holland AT and Palaniappan, LP. Festival Foods in the Immigrant Diet. *J Immigrant Minority Health*. DOI: 10.1007/s10903-012-9705-4. 2012.
32. Joshi, MS, Lamb, R. New foods for old? The diet of South Asians in the UK. Psychol Dev SOc, 2000;12:83-103.
33. Bradby, H. Health, eating and heart attacks: Glasweigian Punjabi women's thinking about everyday food. In Caplan, P (ed.) *Food, Health and Identity.* London: Routledge.1997.
34. Babkus WS and Thompson JL. Physical Activity among South Asian Women: a systematic, mixed methods review. *International Journal of Behavioral Nutrition and Physical Activity,* 2012 9: 150.
35. Jepson, J. et al, 2012 Physical activity in South Asians: an in-depth qualitative study to explore motivations and facilitators.

For peer review only

Figure 1: Trial Summary Consort Flowchart

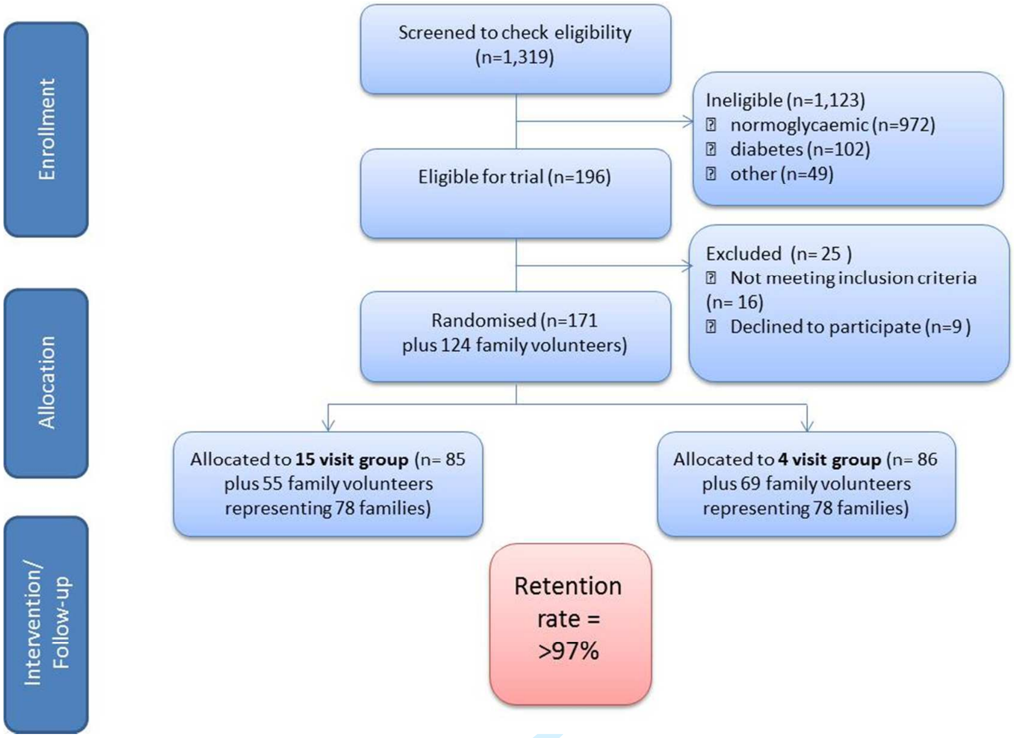

Figure 2: Qualitative study recruitment and data generation

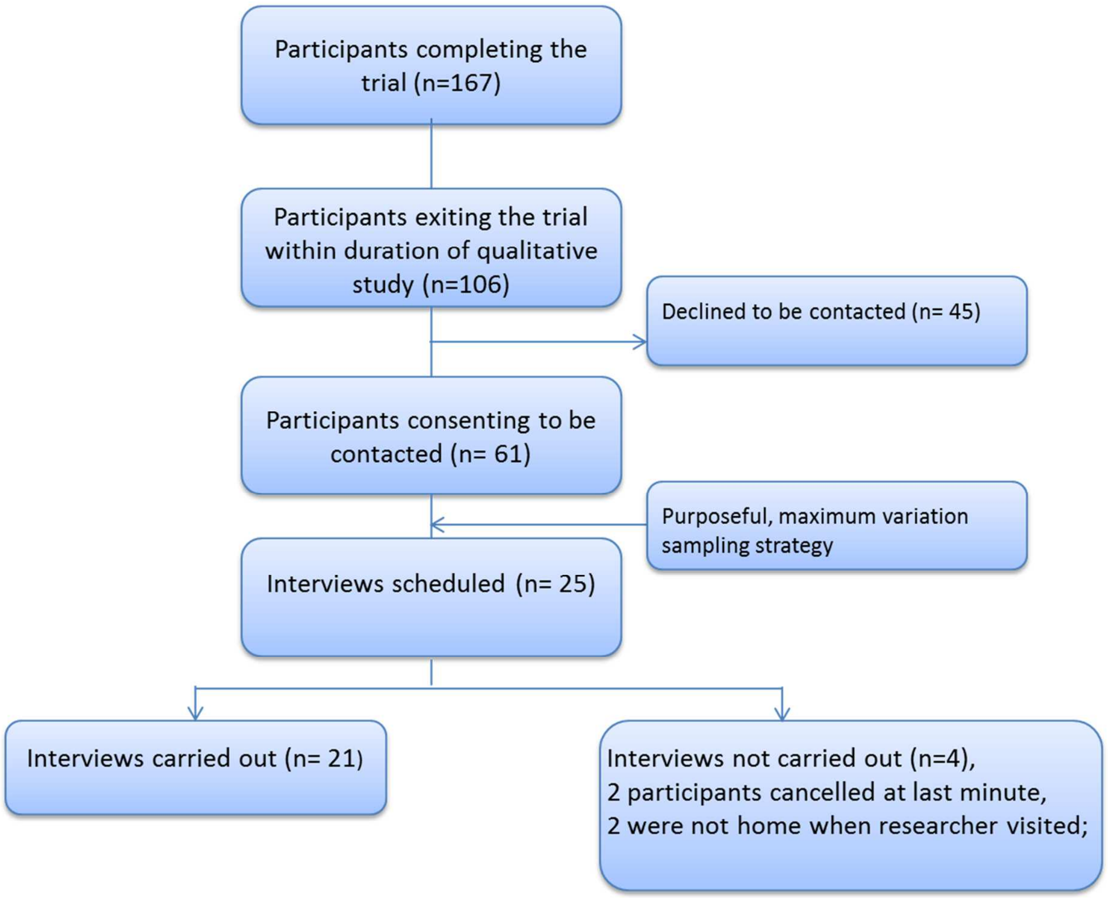

Table 1: Participant population by geographical location

	Glasgow		Edinburgh		Total	
Research subjects	132		39		171	
	Male	Female	Male	Female	Male	Female
	58	74	20	19	78	93
Family volunteers	114		10		124	
	Male	Female	Male	Female	Male	Female
	28	86	0	10	28	96

Table 2: Qualitative study participants by geographical location

	Glasgow		Edinburgh		Total	
Research subjects	14		6		20	
	Male	Female	Male	Female	Male	Female
	9	6	4	2	13	7
Family volunteers	4		0		4	
	Male	Female	Male	Female	Male	Female
	1	3	0	0	1	3

For peer review only

Table 3: Qualitative study participants by intervention, ethnicity, language and faith

	Trial participants	Randomised group		Ethnicity*		Preferred Language*					Faith*			Family Volunteers	
		15 intervention	4 intervention	Indian	Pakistan	English	Urdu	Punjabi	More than one language	Use of interpreter during interview	None	Muslim	Sikh	15 intervention	4 intervention
Male	12	7	5	6	6	9	0	0	3	3	1	6	5	0	1
Female	8	2	6	3	5	3	2	1	2	4		4	4	1	2
Total	20	9	11	9	11	12	2	1	5	7	1	11	9	1	3

*Self-defined

For peer review only

Table 4: Details of interview participants

Interview no.	Location	Randomised group (1=tailored intervention, 2= low intensity intervention)	Gender (M/F)	Ethnicity I=Indian P=Pakistani	Preferred language for speaking	Number of participants (family volunteer)	Researcher
1	Glasgow	2	M	I	Punjabi	1	ZM (and interpreter)
2	Glasgow	2	F	I	Punjabi	1	NC (and interpreter)
3	Edinburgh	1	M	P	English	1	NC
4	Glasgow	2	F	P	Urdu	1	NC
5	Edinburgh	1	F	P	English	1	NC
6	Glasgow	2	F	I	Punjabi	1	NC (and interpreter)
7	Glasgow	2	F	I	Punjabi	(wife)	NC (and interpreter)
8	Glasgow	1	M	I	English	1	NC
9	Edinburgh	2	M	P	English	1	NC
10	Glasgow	1	M	I	English	1	NC
11	Glasgow	2	F	P	Urdu, Hindi, Punjabi	1	NC
12	Glasgow	2	M	I	English	1	NC
13	Glasgow	2	F	P	English	1	NC
14	Edinburgh	1	M	P	English	1	ZM
15	Edinburgh	1	M	I	English	1	ZM
16	Edinburgh	1	F	I	English	1	ZM
17	Glasgow	1	M	P	English	1	ZM
18	Glasgow	1	M	P	Urdu, Punjabi	2 (wife)	ZM (and interpreter)
19	Glasgow	2	M	I	English	2 (brother)	ZM
20	Glasgow	2	M	P	Urdu	1	ZM (and interpreter)
21	Glasgow	2	M	P	English, Urdu	2 (wife)	ZM (and interpreter)

Box 1: Summary of themes within the data

Participation and retention

- Perspectives on potential benefits of the participation;
- Participant choice;
- Relationships.

Adherence to the intervention

- Understanding adherence;
- Reasons for non-adherence

For peer review only - http://bmjopen.bmj.com/site/about/guidelines.xhtml

Understanding experiences of participating in a weight loss lifestyle intervention trial: qualitative evaluation of South Asians at high risk of diabetes

STROBE 2007 (v4) Statement—Checklist of items that should be included in reports of *cross-sectional studies*

Section/Topic	Item #	Recommendation	Reported on page #
**Title and abstract**	1	(*a*) Indicate the study's design with a commonly used term in the title or the abstract	1,2
		(*b*) Provide in the abstract an informative and balanced summary of what was done and what was found	2
**Introduction**			
Background/rationale	2	Explain the scientific background and rationale for the investigation being reported	3,4
Objectives	3	State specific objectives, including any prespecified hypotheses	3
**Methods**			
Study design	4	Present key elements of study design early in the paper	3,4
Setting	5	Describe the setting, locations, and relevant dates, including periods of recruitment, exposure, follow-up, and data collection	3-4
Participants	6	(*a*) Give the eligibility criteria, and the sources and methods of selection of participants	3-4
Variables	7	Clearly define all outcomes, exposures, predictors, potential confounders, and effect modifiers. Give diagnostic criteria, if applicable	4-7
Data sources/ measurement	8*	For each variable of interest, give sources of data and details of methods of assessment (measurement). Describe comparability of assessment methods if there is more than one group	4-7, 12-16
Bias	9	Describe any efforts to address potential sources of bias	4, 13-15
Study size	10	Explain how the study size was arrived at	4-5
Quantitative variables	11	Explain how quantitative variables were handled in the analyses. If applicable, describe which groupings were chosen and why	N/A
Statistical methods	12	(*a*) Describe all statistical methods, including those used to control for confounding	N/A
		(*b*) Describe any methods used to examine subgroups and interactions	
		(*c*) Explain how missing data were addressed	
		(*d*) If applicable, describe analytical methods taking account of sampling strategy	

For peer review only - http://bmjopen.bmj.com/site/about/guidelines.xhtml

Understanding experiences of participating in a weight loss lifestyle intervention trial: qualitative evaluation of South Asians at high risk of diabetes

		(*e*) Describe any sensitivity analyses	
**Results**			
Participants	13*	(a) Report numbers of individuals at each stage of study—eg numbers potentially eligible, examined for eligibility, confirmed eligible, included in the study, completing follow-up, and analysed	2,3-4, 12-17
		(b) Give reasons for non-participation at each stage	3-4
		(c) Consider use of a flow diagram	
Descriptive data	14*	(a) Give characteristics of study participants (eg demographic, clinical, social) and information on exposures and potential confounders	2,3-4, 12-17
		(b) Indicate number of participants with missing data for each variable of interest	2,3-4, 12-17
Outcome data	15*	Report numbers of outcome events or summary measures	2,3, 13
Main results	16	(*a*) Give unadjusted estimates and, if applicable, confounder-adjusted estimates and their precision (eg, 95% confidence interval). Make clear which confounders were adjusted for and why they were included	N/A
		(*b*) Report category boundaries when continuous variables were categorized	
		(*c*) If relevant, consider translating estimates of relative risk into absolute risk for a meaningful time period	
Other analyses	17	Report other analyses done—eg analyses of subgroups and interactions, and sensitivity analyses	4-7
**Discussion**			
Key results	18	Summarise key results with reference to study objectives	5-7
Limitations	19	Discuss limitations of the study, taking into account sources of potential bias or imprecision. Discuss both direction and magnitude of any potential bias	7
Interpretation	20	Give a cautious overall interpretation of results considering objectives, limitations, multiplicity of analyses, results from similar studies, and other relevant evidence	7-9
Generalisability	21	Discuss the generalisability (external validity) of the study results	7-9
**Other information**			
Funding	22	Give the source of funding and the role of the funders for the present study and, if applicable, for the original study on which the present article is based	9

*Give information separately for cases and controls in case-control studies and, if applicable, for exposed and unexposed groups in cohort and cross-sectional studies.

For peer review only - http://bmjopen.bmj.com/site/about/guidelines.xhtml

Understanding experiences of participating in a weight loss lifestyle intervention trial: qualitative evaluation of South Asians at high risk of diabetes

Note: An Explanation and Elaboration article discusses each checklist item and gives methodological background and published examples of transparent reporting. The STROBE checklist is best used in conjunction with this article (freely available on the Web sites of PLoS Medicine at http://www.plosmedicine.org/, Annals of Internal Medicine at http://www.annals.org/, and Epidemiology at http://www.epidem.com/). Information on the STROBE Initiative is available at www.strobe-statement.org.

For peer review only

For peer review only - http://bmjopen.bmj.com/site/about/guidelines.xhtml

BMJ Open

Understanding experiences of participating in a weight loss lifestyle intervention trial: qualitative evaluation of South Asians at high risk of diabetes

Journal:	*BMJ Open*
Manuscript ID:	bmjopen-2013-004736.R1
Article Type:	Research
Date Submitted by the Author:	29-Apr-2014
Complete List of Authors:	Morrison, Zoe; Centre for Population Health Sciences, The University of Edinburgh<br>Douglas, Anne; University of Edinburgh, Centre for Population Health Sciences<br>Bhopal, Raj; University of Edinburgh, Centre for Population Health Sciences<br>Sheikh, Aziz; University of Edinburgh, Division of Community Health Sciences; University of Edinburgh, Centre for Population Health Sciences
**Primary Subject Heading**:	Diabetes and endocrinology
Secondary Subject Heading:	Public health, Qualitative research, Research methods
Keywords:	DIABETES & ENDOCRINOLOGY, General diabetes < DIABETES & ENDOCRINOLOGY, PREVENTIVE MEDICINE, PUBLIC HEALTH, QUALITATIVE RESEARCH

SCHOLARONE™
Manuscripts

Understanding experiences of participating in a weight loss lifestyle intervention trial: qualitative evaluation of South Asians at high risk of diabetes

[1]Zoe Morrison PhD, [1]Anne Douglas MA, [1]Raj Bhopal DSE and [1,2]Aziz Sheikh FRCP on behalf of the trial investigators*

*Trial investigators: John F Forbes, Jason M R Gill, Michael Lean, John McKnight, Gordon Murray, Naveed Sattar, JaakkoTuomilehto and Sarah H Wild

[1]Centre for Population Health Sciences, The University of Edinburgh, Teviot Place, Edinburgh EH8 9AG, UK

[2]Division of General Internal Medicine and Primary Care, Brigham and Women's Hospital/Harvard Medical School, 3rd Floor, 1620 Tremont Street, Boston MA 02120-1613, USA

Corresponding author
Zoe Morrison,
Edinburgh Ethnicity and Health Research Group
Centre for Population Health Sciences,
Medical School, Teviot Place,
Edinburgh EH8 9AG, UK
Telephone: +44 131 651 4147
Fax: +44 131 650 6909
Email: zoe.morrison@ed.ac.uk

Keywords: diabetes mellitus; randomised controlled trial; South Asians; qualitative research, weight control

Abstract

Objective: To explore the reasons for enrolling, experiences of participating and reasons for remaining in a family-based, cluster randomised controlled trial of a dietitian-delivered lifestyle modification intervention aiming to reduce obesity in South Asians at high risk of developing diabetes.

Design: Qualitative study using narrative interviews of a purposive sample of trial participants following completion of the intervention. Data were thematically analysed.

Setting: The intervention was conducted in Scotland and resulted in a modest decrease in weight, but did not statistically reduce the incidence of diabetes.

Participants: We conducted 21 narrative interviews with 24 participants (20 trial participants and four family volunteers).

Results: Many participants were motivated to participate because of: known family history of diabetes and the desire to better understand diabetes related risks to their own and their family's health; ways to mitigate these risks; and to benefit from personalised monitoring. Home-based interventions, communication in the participant's chosen language(s) and continuity in dietitians supported their continuing engagement with the trial. Adaptations in food choices were initially accommodated by participants, although social and faith-based responsibilities were reported as important barriers to persevering with agreed dietary goals. Many participants reported that increasing their level of physical activity was difficult given their long working hours, physically demanding employment and domestic commitments; this being compounded by Scotland's challenging climate and a related reluctance to exercise in the outdoors.

Conclusions: Although participants had strong personal interests in participation and found the information provided by dietitians useful, they nonetheless struggled to incorporate the dietary and exercise recommendations into their daily lives. In particular, increasing levels of physical exercise was described as an additional and in some cases unachievable burden. Consideration needs to be given to strengthening and supporting lifestyle interventions with community-based approaches in order to help overcome wider social and environmental factors.

Article summary

Article focus

- Understanding experiences of participation and reasons for enrolling and remaining in a family-based, cluster randomised controlled trial of a dietitian-delivered lifestyle modification intervention aiming to reduce obesity in South Asians at high risk of developing diabetes.

Key messages

- Participants had strong personal interests in participation and found the information provided by dietitians useful but struggled to incorporate the dietary and exercise recommendations into their daily lives.
- Increasing levels of physical exercise was described as an additional and in some cases unachievable burden.
- We advocate consideration of community-based approaches to help overcome wider social and environmental factors when designing lifestyle interventions.

Strengths and limitations of this study

- We used narrative-based qualitative methods in a culturally appropriate way to understand the social and behavioural dimensions of a culturally adapted randomised lifestyle change trial in Scotland.

- A limitation of this study was the use of interpreters during data generation. We sought to give participants the opportunity to participate in a language of their choosing and could not achieve this without synchronous translation.

For peer review only

Introduction

People of South Asian origin are up to five times more likely than White Scottish adults to develop type II diabetes and, once established, are at particularly high risk of poor outcomes.[1-2] Dietary management and physical activity have been recommended as effective contributors to the prevention of type II diabetes mellitus.[3-7] We undertook an open, family-based, cluster randomised controlled trial ('the trial') that aimed to establish the benefits and cost-effectiveness of a complex dietitian-led dietary- and physical activity-based intervention for reducing obesity and preventing type II diabetes mellitus in people of Indian- and Pakistani-origin at high risk of developing diabetes living in Scotland. Participants were randomised to a tailored intervention, comprising 15 contacts with the trial dietitians over a three year period, or a low-intensity intervention, comprising four visits in the corresponding period.[8]

Cultural adaption of models of care for minority populations is known to be important in promoting equal access to care and self-efficacy[9] and requires inclusion of minority groups in healthcare research.[10] Ensuring participation and retention of these populations in longitudinal studies is a concern.[11,12] Barriers to effective engagement with minority ethnic communities include logistical considerations,[13] language-related barriers,[14] and issues of trust and respect between researchers and marginalised communities.[14,15] Extensive cultural adaptations were built into the trial intervention,[16] specifically flexible home-based interventions by the same dietitian, multilingual written resources and verbal communication, and modification of high-calorie traditional dishes.

The majority of recruitment to the trial was achieved informally through personal communications based upon trusted relationships such as faith-based activities and social groups including lunch clubs, referral by friends and general practitioners, community locations such as specialist food and clothing shops.[12] Participant retention rate within the trial of 97.7% far exceeded expectation. Outcomes of the trial were variable for individual participants. Overall the tailored intervention led to weight loss ≥2·5 Kg for 33 participants compared with 12 in the control group. The intervention group spent 3·0 hrs per week on physical activity compared to the control group's 2·1 hours, with little difference in food shopping and food preparation time. These results are reported in full separately.[17] This paper reports findings from a qualitative study of participant experiences of the trial. As the main trial was a lifestyle intervention utilising quantitative parameters we sought to investigate the social and behavioural aspects of the trial that were not addressed in the main trial outcome measures. We aimed to obtain a rich and multi-faceted understanding of reasons for trial participation and retention, and factors influencing adherence to the lifestyle intervention to give insight into the social and behavioural dimensions of the trial method and results.

Methods

We undertook a qualitative study[18] to inform our understanding of patient experiences of the trial processes.[19] We used narrative-based interviewing,[20] a method that encourages participants to tell their story as they choose, in preference to interviews that utilise structured topic guides. Asking participants to tell their story allows people to spontaneously construct an account of their own experiences in a manner that is strongly shaped by and reflective of their cultural preferences. This method has been found to be an effective method of generating and interpreting experience-centred, culturally orientated data with people of South Asian backgrounds.[21]

The methods for the main trial, including participant inclusion and exclusion criteria, the nature of the randomisation procedure, and retention rate are all described in detail separately.[17] A summary CONSORT flowchart is shown at Figure 1 as contextual information for this study. Details of the trial

For peer review only

participant population are shown in Table 1. We focused upon trial participants and family volunteers exiting the study. We utilised purposeful sampling[22] to ensure representation of the diversity within the trial population by sex, ethnicity, faith group, geographical location (Glasgow and Edinburgh), and whether they were allocated to the intervention or control group.[8]

During their final trial visit, participants were invited by their dietitian to take part in a further piece of related research and given an information sheet regarding the qualitative study. The trial dietitians then obtained consent to be contacted from those who were willing to consider participation. A selection of participants was made utilising the purposeful sampling strategy and contacted to arrange a research meeting, at the beginning of which the qualitative study was explained again before written consent to take part was obtained by the researcher. All interviews were conducted at a location and in the language of the participants' choice; interpreting services were used, if necessary. Interviews lasted between one and two hours and in all but one case took place in the participant's home. Each of the family volunteers who took part was related to one of the 20 trial participants and was interviewed together with their family member. All fieldwork was undertaken with due regard to maintaining the best interests of participants and, in particular, assuring their confidentiality and anonymity. The researcher was blinded to the performance of trial participants throughout the course of the research process, including study design, data generation and analysis.

Narrative methods seek to gather participant accounts of their experiences in their own words in the form of stories by using natural prompts to encourage communication rather than questions to stimulate response.[20] We used preliminary qualitative work (unpublished) to identify open questions to prompt participants' stories, when necessary, including for example:

- What were the motivations, perceptions and attitudes that led you to express an interest and then agree to participate in this trial?
- What factors have influenced your ability to engage with, and be faithful to, the study interventions?
- What factors have influenced participants' decisions to remain involved in the trial?
- More generally, what were the most memorable events and experiences that influenced decisions to accept the invitation for screening, agree to participate, adhere to and remain enrolled in the trial?

Thematic data analysis[20] was concurrently undertaken using NVivio9 software,[23] allowing emerging themes to inform on-going data collection using the constant comparison method.[24] We actively considered alternative explanation cases and allowed for the researcher's reflexive analysis of interpretations.[20] Regular discussions of the emerging findings among the trial qualitative subgroup and the active seeking of disconfirming data further ensured the trustworthiness of findings. Data collection ceased when saturation could reasonably be assumed.[25] Fully anonymised study results were only shared with trial dietitians once the trial was closed.

Results

We conducted 21 narrative interviews with 24 participants (20 trial participants and four family volunteers). We achieved high quality data from a diverse sample, enabling spontaneous account of participants' experiences in their chosen language(s) and narrative structure. Our process of recruitment and data generation is shown in Figure 2. Qualitative study participants are summarised

For peer review only - http://bmjopen.bmj.com/site/about/guidelines.xhtml

by geographical location in Table 2, by intervention, ethnicity, language and faith in Table 3 and detailed in full in Table 4. Findings are reported in conjunction with illustrative data in relation to experiences of participation and retention within the trial, and adherence to the intervention. Our analytical framework is summarised in Box 1. We did not identify significant points of differentiation between the intervention and control group participants. We did identify a small number of gender-specific considerations.

Participation and retention in the trial

Perceived benefits of participation in the trial, the accommodation of participant choice of language and location, and trusting relationships between trial investigators and participants contributed to the trial's very high retention rate.

Perspectives on potential benefits of participation

Participants could not always fully recall how they were recruited into the trial. Two participants described themselves as having joined the trial because they were *'on the borderline'* of having diabetes (I.15,18) and three specifically noted their desire to lose weight (I.8,13,19).Some participants had been attracted by the provision of health related information (I.3,5,6,7,10,12). Increased awareness was particularly attractive in the light of family histories of diabetes mellitus (I.4,10,13,14,15,17,19,11).

> *"My parents had diabetes and my father-in-law also had, so I have always had fear about it. In fact I consulted libraries also to gain knowledge about it."* (I.11)

Other participants were in contrast more ambivalent about the trial, participating on the basis of *'no harm'* rather than in pursuit of direct benefit (I.1,6,9).

Participant choice

The emphasis upon patient choice built into the trial design contributed to participant engagement and retention. Accommodating preferences for consultation at home or in clinic was appreciated as some welcomed the opportunity to get out (I.10,15) whilst others welcomed home consultations (I.4,9,17). However, not every participant kept a diary and home visits presented some scheduling difficulties that required out-of-hours working by dietitians, as one participant observed:

> *"You can't do it all the time because the people are at home at different times of the day, some are in the evening, some late evening, they are not there during the day time."* (I.9)

Being able to speak to female dietitians in their language(s) of choice was valued by female participants (I.4,7,11,13), with participants feeling free to take the approach best suited to them:

> *"It made me – like – relaxed when she was around because I didn't, sometimes I don't understand in English, then I could speak to her in Indian. But mostly I didn't want to speak to her in Indian."* (I.16)

Such preferences were less frequently expressed by men (I.18,19).

Relationships

Some participants decided to participate in the trial as a result of knowing members of the research team. In addition, the relationships that developed between participants and their dietitians over the course of the trial positively contributed to retention. Whilst participants did not commonly refer to or produce for discussion their written materials, they placed importance on good verbal communication (I.11,16), empathy and compassion (I.7,10,18).

> *"She used to advise me but so compassionately and afterwards I used to think over it that it is for my help ultimately I will be the one who will benefit she is not my relative still she cares so much."* (I.19)

Adherence to the intervention

Adherence to the trial proved a complex issue to investigate as participants were well-informed and reported the correct behaviours even if they were not pursuing these. Adherence involved different understandings of the advocated lifestyle changes and unanticipated motivations such as caring for other family members or a sense of obligation to the dietitian. Non-adherence to the trial was in some case culturally motivated, or due to contextual factors such as climate or lack of time.

Understanding adherence

Participants who felt they had adhered to the trial mostly described changes they had made in relation to frequency of exercise (I.11.12) and more informed food choices (I.4,10,11,17,18,21). Some felt that this had not been a particularly significant change as they had a balanced diet already (I.9,12). Conversely, some were evangelical about the trial and their related achievements. Some had lost significant amounts of weight (e.g. *"half a stone"* (I.11)). Two gentleman described how their lives had fundamentally changed, one having given up high alcohol consumption (I.15), the other having become a runner for charity:

> *"I could have never imagined ...I could not even walk 2 kilometres then I have started doing 5 [km] then 10 [km] then I have done lot of 10 then I thought lets go for the bigger [half marathon]then it took me 2hours and 19 minutes ...So with that time we raised £2,000 - I was in the paper!"* (I.19)

Many participants had difficulty in sustaining lifestyle changes (I.10,16,13,14) describing a process of *"fits and starts"* (I.10) as they struggled consistently to follow the advice given despite known health risks. This was true even subsequent to a diagnosis of diabetes (I.7,16). In contrast, looking after other family members influenced adherence. One participant attributed a weight loss of 8kg in part to different methods of food preparation (e.g. less frying) and their having increased their daily exercise due to the need to look after their son's dog (I.8). Another described how they had followed the intervention because they felt it would be beneficial to the whole family (I.11).

Scheduled consultations themselves impacted on levels of adherence as participants either avoided their appointments as they were on the *"downward slope"* (I.10,11) or adapted their behaviour in anticipation:

> *"She used to come in every three months so before she used to come I used to feel a kind of pressure to make sure that my weight had not increased."* (I.18)

Reasons for non-adherence

Culture considerations were evident in discussions regarding the challenges of adherence to the lifestyle intervention. The importance of food within South Asian cultures was referred to for example in the context of sharing home-made sweets with grandchildren (I.2,14) and traditional food preparation techniques:

> *"Once a week they have children all come so we feel that the food should be much nicer according to the tradition and also children don't like ordinary vegetables they fancy food like from McDonald's so just to compete with that kind of food we try to make our old Indo-Pakistani dishes."* (I.20)

The role of food in community functions (I.15) and faith were also given as challenges to adherence, with one participant feeling she couldn't avoid gaining weight:

> *"I know that if I lose more weight I'm going to put it back on at Ramadan because I just have to walk by food and it just adds onto my body, the fat just is invisible and it runs after you."* (I.13)

Whilst the trial was designed to be culturally sensitive to the needs of South Asian populations, some members of the population felt that the advice did not accommodate their more international food preferences (I.10,13,15).

Other reasons for non-adherence related to wider contextual factors. Participants felt they were too busy to find the time to exercise (I.1,4,14,15,16). Two male participants noted the physical demands of their employment as a barrier to exercise (I.14,17), whilst some females felt unable to get enough time out of the house (I.2):

> *"I try to go for walks but it's hard to find time as I have so many grand-children and women in our community don't get out of the house that much. There is no one else who will come with me."* (I.4)

The climate was a consideration for South Asians living in Scotland as participants found recommended exercise such as walking problematic in the cold (I.1,2,15,16) and food was described as a cultural representation of warmth (I.2). It therefore seemed counter-intuitive to increase one without the other.

Discussion

We used qualitative methods to understand the social and behavioural dimensions of the first family-based, culturally adapted randomised lifestyle change trial to investigate approaches to reducing obesity and prevent type II diabetes mellitus in people of Indian- and Pakistani-origin living in Scotland. Findings indicate that individuals may not necessarily prioritise their own health over other factors when making lifestyle choices despite known risks and prevention strategies. Participants were attracted to the trial by the availability of regular health monitoring and personalised information, particularly those who were aware of a family history of diabetes. The choice of home- or clinic-based interventions, communication in the participants' chosen language(s) and trusting relationships between participants and dietitians contributed to the high retention rate. Participants stated that they did not find adherence to advice on food choices and preparation problematic, although some conceded that they did not always choose to take the advice, citing examples of community and faith based considerations that made consistent adherence difficult. Participants did find increasing their levels of physical activity problematic given demands such as the Scottish climate, long working hours, physically demanding jobs and domestic commitments.

One important limitation of this study was the use of interpreters during data generation. We sought to give participants the opportunity to participate in a language of their choosing and could not achieve this without synchronous translation. During the initial stages of the study retrospective quality checking of interview audio and transcripts highlighted that the interpreter was not accurately reporting the views of the participants. We addressed this by recruiting a new interpreter and providing training as to the exact nature and purpose of the services required. To ensure that the data

For peer review only

from the three affected interviews were not lost we had the original audio recording re-translated. We note however that the conduct of these interviews was compromised to a small extent by this limitation. A further limitation is the lack of inclusion of Indian Hindu participants and the small sample of family volunteers. For practical reasons (such as interview cancellation by participants) we could not achieve this within our sample during the time available.

In discussing our findings in the light of current literature on participation and retention we consider the benefits sought from the intervention, flexibility within the trial design and participant/dietitian relationships. Regarding adherence our participants talked about lifestyle changes and we have reported findings accordingly. In contrast, the literature considers attitudes to food and physical activity separately and we discuss our findings in this context.

The need for individualised and context specific information for people with pre-diabetes has been acknowledged[26] and was a benefit participants sought from engaging with the trial. People recognised they were at risk due to family histories. Losing weight was not articulated as a dominant sought-after benefit even though it was a secondary outcome of the trial and this may be due to culturally situated traditional beliefs about body size. In South Asian communities a tendency to equate large size with healthy womanhood, marriage and reproduction, securing a good job and high social status has been identified,[27] although this view may be less prevalent now. Understanding of the causal relationship between weight and diabetes was less evident, re-iterating calls for education within South Asian communities on the causes and prevention of diabetes.[28]

Findings highlight the importance of accommodating participant choice in the design of complex lifestyle interventions. Appreciation of flexibility within diabetes prevention programmes has been recognised,[29] as has the need to overcome known barriers to effectively engaging with minority ethnic communities.[13-15] Whilst culturally sensitive adaptations are essential and were achieved within this trial[16] they should avoid reinforcing stereotypes,[52] for example by focusing on traditional dishes. This did not necessarily sufficiently accommodate complex patterns of dietary acculturation.[31]

Despite cultural adaptation, the trial written resources were rarely referred to. In contrast, verbal communication and participant/dietitian relationships were repeatedly referred to. The importance of non-judgemental professional attitudes, and empathy within self-management interventions has been recognised[26,29] and, together with known issues of trust and respect between researchers and marginalised communities, [14,15] was anticipated in the trial design. The intimate nature of this home based lifestyle intervention required a compassionate approach to investigation, out-of-hours working and an emphasis on participant choice different to that of the majority of interventions delivered in clinical settings. Dietitian skill in building relationships was felt to be very important in addition to skills in the verbal communication of advice and information. The importance of continuity in dietician staff was emphasised in trial design, but the strength of participant-dietitian relationships was not anticipated and proved to be significant in retention across the trial (99% in intervention group and 97% in control group). In some cases it seemed that participant loyalty was to the dietitian rather than the trial. This indicates the importance of relational aspects of trial participation that would benefit from further investigation.

Our findings suggest that the cultural construction of food made it difficult for South Asians to make the healthy food choices advised despite known health risks. For members of this community, food is the focus of good living and etiquette.[27] In communities that have previously faced economic insecurity, being able to afford food is a sign of success, demonstrating social power and hospitality.[27,31,32]Although most migrants alter their eating habits following migration,[31] South Asian

For peer review only - http://bmjopen.bmj.com/site/about/guidelines.xhtml

systemic beliefs regarding the heating and cooling properties and merits of food endure.[33] The role of food in community- and faith-based activities together with complex intergenerational patterns of dietary acculturation detracted from self-reported consistent adherence to both the tailored and low-intensity interventions.

It is known that the South Asian population has lower levels of physical activity than the general population and lesser intrinsic motivations for physical exercise.[34,35] We have corroborated proposed reasons for this, including lack of time and/or childcare[34,35] and gendered barriers to exercise, such as the availability of single-sex facilities and the depiction of exercise as a selfish act detracting from family care.[35] We further note the role of climate and the interplay between South Asian systemic beliefs regarding the heating and cooling properties and merits of food[33]and the demands of living and undertaking physical activity in the cold Scottish climate.

Our study considered one research sub-population: the South Asian community living in Scotland. A more general insight from these findings is that well-informed individuals may not necessarily choose or feel able to act on health-related advice due to other lifestyle considerations. People sought health related information as the main benefit of participation and the dietary requirements of the trial were not felt by participants to be an extra burden in their everyday life. However, culturally situated influences on food choices often took precedence over the advice and information given in both intervention and control groups despite acknowledged health implications. This highlights the need for the adaptation of lifestyle interventions to accommodate cultural considerations amongst ethnic minority research participants.

The increased levels of physical activity were however seen to pose an additional burden by participants, again due to cultural factors including those previously identified, such as lower intrinsic motivation, a lack of time and/or access to appropriate facilities, and domestic considerations, each and all of which may impact upon the prevention and management of diabetes In addition, findings highlighted the role of climate in decisions relating to food and physical activity. This suggests that diet and exercise are less distinct within South Asian communities and indicates a need to better understand the impact of climate on lifestyle choices for immigrant populations.

For peer review only

Acknowledgements: We have previously thanked and named many individuals who contributed to the trial.(8) We further acknowledge the Trial Dietitians (Alyson Hutchison, Anu Sharma, Sunita Wallia and Ruby Bhopal), Nandini Choudhuri, Sujama Roy and Salman Aziz; current Trial Steering Committee (Prof Nigel Unwin (ex-chair), Prof Graham Hitman (chair), Dr Nita Forouhi, Dr Deepak Bhatnagar, Dr Ghada Zoubiane , and Mr Iqbal Anwar); the trial Data Monitoring and Ethics Committee (Prof Iain Crombie, Dr Mike Small, Dr Mike Kelly, Prof Kamlesh Khunti); South Asian Community & religious organisations and individuals who contributed to the recruitment efforts; and finally to all the participants who gave their time to take part in this study.

Funding: This study was funded by the National Prevention Research Initiative (G0501310), a funding consortium comprising the British Heart Foundation; Cancer Research UK; Department of Health; Diabetes UK; Economic and Social Research Council; Medical Research Council; Health & Social Care Research & Development Office for Northern Ireland; Chief Scientist Office, Scottish Government Health Directorate; the Welsh Assembly Government; and World Cancer Research Fund. Additional financial support was provided from NHS Lothian and NHS Greater Glasgow & Clyde R&D, Chief Scientist Office, NHS Health Scotland and NHS National Services Scotland.

Contributors: Zoe Morrison and Aziz Sheikh were responsible for the study design, supervised the data collection, and drafted the manuscript. Anne Douglas and Raj Bhopal designed the protocol for the larger study and assisted with obtaining consent for data collection together with other members of the trial team. Each of the authors was involved in data analysis and interpretation, critical review and refinement of the manuscript.

Competing interests: We declare that we have no conflict of interest.

Data sharing statement: Interview audio recordings and transcripts are available from the corresponding author at the University of Edinburgh, who will provide a permanent, citable and open access home for the dataset.

FIGURE LEGENDS:

Figure 1: Main trial summary consort flowchart

Figure 2: Qualitative study recruitment and data generation

For peer review only

References

1. Fischbacher C. M., Bhopal R., Steiner M., et al. Is there equity of service delivery and intermediate outcomes in South Asians with type 2 diabetes? Analysis of DARTS database and summary of UK publications. *Journal of Public Health*, 2009; 31: 2; 239 – 249. doi 10.1093/pubmed/fdp003.
2. Worth A, Irshad T, Bhopal R, et al. Vulnerability and access to care for South Asian Sikh and Muslim patients with life limiting illness in Scotland: prospective longitudinal qualitative study. *British Medical Journal,* 2009; 338: b183. doi: 10.1136/bmj.b183.
3. Li G, Zhang P, Wang J, et al. The long-term effect of lifestyle interventions to prevent diabetes in the China Da Qing Diabetes Prevention Study: a 20-year follow-up study. *Lancet* 2008 May 24;371(9626):1783-9.
4. Tuomilehto J, Lindstrom J, Eriksson JG, et al. Prevention of type 2 diabetes mellitus by changes in lifestyle among subjects with impaired glucose tolerance. *N Engl J Med* 2001 May 3;344(18):1343-50.
5. Knowler WC, Barrett-Connor E, Fowler SE, et al. Reduction in the incidence of type 2 diabetes with lifestyle intervention or metformin. *New England Journal of Medicine* 2002 Feb 7;346(6):393-403.
6. Gillies CL, Abrams KR, Lambert PC, et al. Pharmacological and lifestyle interventions to prevent or delay type 2 diabetes in people with impaired glucose tolerance: systematic review and meta-analysis. *BMJ* 2007 Feb 10;334(7588):299.
7. Hopper I, Billah B, Skiba M, et al. Prevention of diabetes and reduction in major cardiovascular events in studies of subjects with prediabetes: meta-analysis of randomised controlled clinical trials. *Eur J Cardiovasc Prev Rehabil* 2011 Dec;18(6):813-23.
8. Douglas A, Bhopal RS, Bhopal R, et al. Design and baseline characteristics of the PODOSA (Prevention of Diabetes & Obesity in South Asians) trial: a cluster, randomised lifestyle intervention in Indian and Pakistani adults with impaired glycaemia at high risk of developing type 2 diabetes. *BMJ Open*, 2013; Feb 22;3(2). pii: e002226. doi: 10.1136/bmjopen-2012-002226.
9. Griffiths, C, Motlib, J, Azad,A et al. Randomised controlled trial of a lay-led self-management programme for Bangladeshi patients with chronic disease. *Br J Gen Pract.* 2005 November 1; 55(520): 831–837.
10. Mason S, Hussain-Gambles M, Leese B, et al. Representation of South Asian people in randomised clinical trials: analysis of trials' data. *BMJ* 2003; 326: 1244–1245.
11. Sheikh A, Netuveli G, Kai J, et al. Comparison of reporting of ethnicity in US and European randomised controlled trials. *BMJ* 2004; 329: 87-8.
12. Samsudeen B S, Douglas A, Bhopal R. Challenges in recruiting South Asians into prevention trials; health professional and community recruiters' perceptions on the PODOSA trial. *Public Health* 2011; 125:201-209.
13. Hussain-Gambles M. South Asian patients' views and experiences of clinical trial participation. *Family Practice* 2004; 21: 636-42.
14. Stirland L, Halani L, Bhopal, R, et al. Recruitment of South Asians into asthma research: qualitative study of UK and US researchers. *Primary Care Respir J.* 2011 Sep;20(3):282-90. Doi:10.4104/pcrj.2011.00032
15. Harris, Y, Gorelick PB, Samuels P, et al. Why African Americans may not be participating in clinical trials. *J Natl Med Assoc* 1996: 88: 630-634.

For peer review only

16. Wallia S, Bhopal R.S., Douglas A, et al. Culturally adapting the prevention of diabetes and obesity in South Asians (PODOSA) trial. *Health Promot Int* 2013
17. Bhopal RS, Douglas A, Walia S, et al. Weight change over three-years in South Asians at high risk of diabetes: effects and costs in a cluster-randomised trial of a lifestyle intervention. *Lancet Diab Endocrin 2014:* 2: 218-27.
18. Malterud K. Qualitative Research: standards, challenges and guidelines. *Lancet*, 2001:358; 483-488.
19. The PLOS Medicine Editors. Qualitative research: understanding patients' needs and experiences. *PLoS Med* 4(8): e258.doi:10.1371/journal.pmed.0040258
20. Riessman C K. *Narrative Methods for the Human Sciences,* London: Sage; 2008.
21. Squire C. Experience-centred and culturally orientated approaches to narrative. In: Andrews M, Quire C, and Tamboukou M. (eds.) *Doing Narrative Research,* London: Sage; 2008.
22. Patton MQ. (1990) *Qualitative evaluation and research methods.* 2nd ed. London: Sage Publications.
23. QSR International Pty Ltd. NVivo qualitative data analysis software Version 9. 2008.
24. Barbour, R. *Introducing Qualitative Research.* Sage: London; 2008.
25. Morse J. The significance of saturation. *Qualitative Health Research.* 1995;5(2):147-
26. Maindal et al. Lifting the lid of the "black intervention box" –the systematic development of an action competence programme for people with screen-detected dysglycaemia. *BMC Health Services Research* 2010,10: 114.
27. Bush, HM, Williams, RGA, Lean, MEJ et al. Body image and weight consciousness among South Asian, Italian and general population women in Britain. *Appetite*, 2001, 37:207-215.
28. Rankin, R and Bhopal, R. Understanding of heart disease and diabetes in a South Asian community: cross-sectional study tsting the 'snowball' sample method. *Public Health*, 2001; 253-260.
29. Walker, C, Hernan, A, Reddy, et al. "Sustaining modified behaviours learnt in a diabetes prevention program in regional Australia: the role of social context. *BMC Health Services Research* 2012, 12: 460.
30. Liamputtong, P. *Researching the vulnerable.* Sage: London; 2007.
31. Azar, KMJ, Chen, E. Holland AT, et al. Festival Foods in the Immigrant Diet. *J Immigrant Minority Health*. DOI: 10.1007/s10903-012-9705-4. 2012.
32. Joshi, MS, Lamb, R. New foods for old? The diet of South Asians in the UK. Psychol Dev SOc, 2000;12:83-103.
33. Bradby, H. Health, eating and heart attacks: Glasweigian Punjabi women's thinking about everyday food. In Caplan, P (ed.) *Food, Health and Identity.* London: Routledge.1997.
34. Babkus WS and Thompson JL. Physical Activity among South Asian Women: a systematic, mixed methods review. *International Journal of Behavioral Nutrition and Physical Activity,* 2012, 9: 150.
35. Jepson, J. et al, 2012 Physical activity in South Asians: an in-depth qualitative study to explore motivations and facilitators.

For peer review only

Box 1: Summary of themes within the data

Participation and retention

- Perspectives on potential benefits of the participation;
- Participant choice;
- Relationships.

Adherence to the intervention

- Understanding adherence;
- Reasons for non-adherence

For peer review only

Table 1: Participant population by geographical location

	Glasgow		Edinburgh		Total	
Research subjects	132		39		171	
	Male	Female	Male	Female	Male	Female
	58	74	20	19	78	93
Family volunteers	114		10		124	
	Male	Female	Male	Female	Male	Female
	28	86	0	10	28	96

Table 2: Qualitative study participants by geographical location

	Glasgow		Edinburgh		Total	
Research subjects	14		6		20	
	Male	Female	Male	Female	Male	Female
	9	6	4	2	13	7
Family volunteers	4		0		4	
	Male	Female	Male	Female	Male	Female
	1	3	0	0	1	3

Table 3: Qualitative study participants by intervention, ethnicity, language and faith

	Trial participants	Randomised group		Ethnicity*		Preferred Language*					Faith*			Family Volunteers	
		15 intervention	4 intervention	Indian	Pakistan	English	Urdu	Punjabi	More than one language	Use of interpreter during interview	None	Muslim	Sikh	15 intervention	4 intervention
Male	12	7	5	6	6	9	0	0	3	3	1	6	5	0	1
Female	8	2	6	3	5	3	2	1	2	4		4	4	1	2
Total	20	9	11	9	11	12	2	1	5	7	1	11	9	1	3

*Self-defined

For peer review only

Table 4: Details of interview participants

Interview no.	Location	Randomised group (1=tailored intervention, 2= low intensity intervention)	Gender (M/F)	Ethnicity I=Indian P=Pakistani	Preferred language for speaking	Number of participants (family volunteer)	Researcher
1	Glasgow	2	M	I	Punjabi	1	ZM (and interpreter)
2	Glasgow	2	F	I	Punjabi	1	NC (and interpreter)
3	Edinburgh	1	M	P	English	1	NC
4	Glasgow	2	F	P	Urdu	1	NC
5	Edinburgh	1	F	P	English	1	NC
6	Glasgow	2	F	I	Punjabi	1	NC (and interpreter)
7	Glasgow	2	F	I	Punjabi	(wife)	NC (and interpreter)
8	Glasgow	1	M	I	English	1	NC
9	Edinburgh	2	M	P	English	1	NC
10	Glasgow	1	M	I	English	1	NC
11	Glasgow	2	F	P	Urdu, Hindi, Punjabi	1	NC
12	Glasgow	2	M	I	English	1	NC
13	Glasgow	2	F	P	English	1	NC
14	Edinburgh	1	M	P	English	1	ZM
15	Edinburgh	1	M	I	English	1	ZM
16	Edinburgh	1	F	I	English	1	ZM
17	Glasgow	1	M	P	English	1	ZM
18	Glasgow	1	M	P	Urdu, Punjabi	2 (wife)	ZM (and interpreter)
19	Glasgow	2	M	I	English	2 (brother)	ZM
20	Glasgow	2	M	P	Urdu	1	ZM (and interpreter)
21	Glasgow	2	M	P	English, Urdu	2 (wife)	ZM (and interpreter)

only

1

Understanding experiences of participating in a weight loss lifestyle intervention trial: qualitative evaluation of South Asians at high risk of diabetes

[1]Zoe Morrison PhD, [1]Anne Douglas MA, [1]Raj Bhopal DSE and [1,2]Aziz Sheikh FRCP on behalf of the trial investigators*

*Trial investigators: John F Forbes, Jason M R Gill, Michael Lean, John McKnight, Gordon Murray, Naveed Sattar, JaakkoTuomilehto and Sarah H Wild

[1]Centre for Population Health Sciences, The University of Edinburgh, Teviot Place, Edinburgh EH8 9AG, UK

[2]Division of General Internal Medicine and Primary Care, Brigham and Women's Hospital/Harvard Medical School, 3rd Floor, 1620 Tremont Street, Boston MA 02120-1613, USA

Corresponding author
Zoe Morrison,
Edinburgh Ethnicity and Health Research Group
Centre for Population Health Sciences,
Medical School, Teviot Place,
Edinburgh EH8 9AG, UK
Telephone: +44 131 651 4147
Fax: +44 131 650 6909
Email: zoe.morrison@ed.ac.uk

Keywords: diabetes mellitus; randomised controlled trial; South Asians; qualitative research, weight control

Abstract

Objective: To explore the reasons for enrolling, experiences of participating and reasons for remaining in a family-based, cluster randomised controlled trial of a dietitian-delivered lifestyle modification intervention aiming to reduce obesity in South Asians at high risk of developing diabetes.

Design: Qualitative study using narrative interviews of a purposive sample of trial participants following completion of the intervention. Data were thematically analysed.

Setting: The intervention was conducted in Scotland and resulted in a modest decrease in weight, but did not statistically reduce the incidence of diabetes.

Participants: We conducted 21 narrative interviews with 24 participants (20 trial participants and four family volunteers).

Results: Many participants were motivated to participate because of: known family history of diabetes and the desire to better understand diabetes related risks to their own and their family's health; ways to mitigate these risks; and to benefit from personalised monitoring. Home-based interventions, communication in the participant's chosen language(s) and continuity in dietitians supported their continuing engagement with the trial. Adaptations in food choices were initially accommodated by participants, although social and faith-based responsibilities were reported as important barriers to persevering with agreed dietary goals. Many participants reported that increasing their level of physical activity was difficult given their long working hours, physically demanding employment and domestic commitments; this being compounded by Scotland's challenging climate and a related reluctance to exercise in the outdoors.

Conclusions: Although participants had strong personal interests in participation and found the information provided by dietitians useful, they nonetheless struggled to incorporate the dietary and exercise recommendations into their daily lives. In particular, increasing levels of physical exercise was described as an additional and in some cases unachievable burden. Consideration needs to be given to strengthening and supporting lifestyle interventions with community-based approaches in order to help overcome wider social and environmental factors.

Article summary

Article focus

- Understanding experiences of participation and reasons for enrolling and remaining in a family-based, cluster randomised controlled trial of a dietitian-delivered lifestyle modification intervention aiming to reduce obesity in South Asians at high risk of developing diabetes.

Key messages

- Participants had strong personal interests in participation and found the information provided by dietitians useful but struggled to incorporate the dietary and exercise recommendations into their daily lives.
- Increasing levels of physical exercise was described as an additional and in some cases unachievable burden.
- We advocate consideration of community-based approaches to help overcome wider social and environmental factors when designing lifestyle interventions.

Strengths and limitations of this study

- We used ~~family~~narrative-based qualitative methods in a culturally appropriate way to understand the social and behavioural dimensions of a culturally adapted randomised lifestyle change trial in Scotland.

- A limitation of this study was the use of interpreters during data generation. We sought to give participants the opportunity to participate in a language of their choosing and could not achieve this without synchronous translation.

For peer review only

Introduction

People of South Asian origin are up to five times more likely than White Scottish adults to develop type II diabetes and, once established, are at particularly high risk of poor outcomes.[1-2] Dietary management and physical activity have been recommended as effective contributors to the prevention of type II diabetes mellitus.[3-7] We undertook an open, family-based, cluster randomised controlled trial (‘the trial’) that aimed to establish the benefits and cost-effectiveness of a complex dietitian-led dietary- and physical activity-based intervention for reducing obesity and preventing type II diabetes mellitus in people of Indian- and Pakistani-origin at high risk of developing diabetes living in Scotland. Participants were randomised to a tailored intervention, comprising 15 contacts with the trial dietitians over a three year period, or a low-intensity intervention, comprising four visits in the corresponding period.[8]

Cultural adaption of models of care for minority populations is known to be important in promoting equal access to care and self-efficacy[9] and requires inclusion of minority groups in healthcare research.[10] Ensuring participation and retention of these populations in longitudinal studies is a concern.[11,12] Barriers to effective engagement with minority ethnic communities include logistical considerations,[13] language-related barriers,[14] and issues of trust and respect between researchers and marginalised communities.[14,15] Extensive cultural adaptations were built into the trial intervention,[16] specifically flexible home-based interventions by the same dietitian, multilingual written resources and verbal communication, and modification of high-calorie traditional dishes.

The majority of recruitment to the trial -was achieved informally through personal communications based upon trusted relationships such as faith-based activities and social groups including lunch clubs, referral by friends and general practitioners, community locations such as specialist food and clothing shops.[12] Participant retention rate within the trial of 97.7% far exceeded expectation. Outcomes of the trial were variable for individual participants. Overall the tailored intervention led to weight loss ≥2·5 Kg for 33 participants compared with 12 in the control group. The intervention group spent 3·0 hrs per week on physical activity compared to the control group’s 2·1 hours, with little difference in food shopping and food preparation time. These results are reported in full separately.[17] This paper reports findings from a qualitative study of participant experiences of the trial. As the main trial was a lifestyle intervention utilising quantitative parameters we sought to investigate the social and behavioural aspects of the trial that were not addressed in the main trial outcome measures. We aimed to obtain a rich and multi-faceted understanding of reasons for trial participation and retention, and factors influencing adherence to the lifestyle intervention to give insight into the social and behavioural dimensions of the trial method and results.

Methods

We undertook a qualitative study[18] to inform our understanding of patient experiences of the trial processes.[19] We used narrative-based interviewing,[20] a method that encourages participants to tell their story as they choose, in preference to interviews that utilise structured topic guides. Asking participants to tell their story allows people to spontaneously construct an account of their own experiences in a manner that is strongly shaped by and reflective of their cultural preferences. This method has been found to be an effective method of generating and interpreting experience-centred, culturally orientated data with people of South Asian backgrounds.[21]

The methods for the main trial, including participant inclusion and exclusion criteria, the nature of the randomisation procedure, and retention rate are all described in detail separately.[17] A summary CONSORT flowchart is shown at Figure 1 as contextual information for this study. Details of the trial

Formatted: Not Superscript/ Subscript

Formatted: Font: (Default) Times New Roman

Formatted: Font: (Default) Times New Roman

Formatted: Font: (Default) Times New Roman

Formatted: Superscript

For peer review only

participant population are shown in Table 1. We focused upon trial participants and family volunteers exiting the study. We utilised purposeful sampling[22] to ensure representation of the diversity within the trial population by sex, ethnicity, faith group, geographical location (Glasgow and Edinburgh), and whether they were allocated to the intervention or control group.[8]

During their final trial visit, participants were invited by their dietitian to take part in the a further piece of related research qualitative study and given an information sheet regarding the qualitative study. The trial dietitians then obtained consent to be contacted from those who were willing to consider participation. A selection of participants -wasere made utilising the purposeful sampling strategy and then contacted to arrange a research meeting, at the beginning of which the qualitative study was explained again before written consent to take part was obtained by the researcher. All interviews were -conducted at a location and in the language of the participants' choice; interpreting services were used, if necessary. Interviews lasted between one and two hours and in all but one case took place in the participant's home. Each of the family volunteers who took part was related to one of the 20 trial participants and was interviewed together with their family member. All fieldwork was undertaken with due regard to maintaining the best interests of participants and, in particular, assuring their confidentiality and anonymity. The researcher was blinded to the performance of trial participants throughout the course of the research process, including study design, data generation and analysis.

Narrative methods seek to gather participant accounts of their experiences in their own words in the form of stories by using natural prompts to encourage communication rather than questions to stimulate response.[20] We used preliminary qualitative work (unpublished) to identify open questions to prompt participants' stories, when necessary, including for example:

Formatted: Superscript

- What were the motivations, perceptions and attitudes that led you to express an interest and then agree to participate in this trial?
- What factors have influenced your ability to engage with, and be faithful to, the study interventions?
- What factors have influenced participants' decisions to remain involved in the trial?
- More generally, what were the most memorable events and experiences that influenced decisions to accept the invitation for screening, agree to participate, adhere to and remain enrolled in the trial?

Thematic data analysis[20] was concurrently undertaken using NVivio9 software,[23] allowing emerging themes to inform on-going data collection using the constant comparison method.[24] We actively considered alternative explanation cases and allowed for the researcher's reflexive analysis of interpretations.[20] Regular discussions of the emerging findings among the trial qualitative subgroup and the active seeking of disconfirming data further ensured the trustworthiness of findings. Data collection ceased when saturation could reasonably be assumed.[25] Fully anonymised study results were only shared with trial dietitians once the trial was closed.

Results

We conducted 21 narrative interviews with 24 participants (20 trial participants and four family volunteers). We achieved high quality data from a diverse sample, enabling spontaneous account of participants' experiences in their chosen language(s) and narrative structure. Our process of

For peer review only - http://bmjopen.bmj.com/site/about/guidelines.xhtml

recruitment and data generation is shown in Figure 2. Qualitative study participants are summarised by geographical location in Table 2, by intervention, ethnicity, language and faith in Table 3 and detailed in full in Table 4. Findings are reported in conjunction with illustrative data in relation to experiences of participation and retention within the trial, and adherence to the intervention. Our analytical framework is summarised in Box 1. We did not identify significant points of differentiation between the intervention and control group participants. We did identify a small number of gender-specific considerations.

Participation and retention in the trial

Perceived benefits of participation in the trial, the accommodation of participant choice of language and location, and trusting relationships between trial investigators and participants contributed to the trial's very high retention rate.

Perspectives on potential benefits of participation

Participants could not always fully recall how they were recruited into the trial. Two participants described themselves as having joined the trial because they were *'on the borderline'* of having diabetes (I.15,18) and three specifically noted their desire to lose weight (I.8,13,19).Some participants had been attracted by the provision of health related information (I.3,5,6,7,10,12). Increased awareness was particularly attractive in the light of family histories of diabetes mellitus (I.4,10,13,14,15,17,19,11).

> *"My parents had diabetes and my father-in-law also had, so I have always had fear about it. In fact I consulted libraries also to gain knowledge about it."* (I.11)

Other participants were in contrast more ambivalent about the trial, participating on the basis of *'no harm'* rather than in pursuit of direct benefit (I.1,6,9).

Participant choice

The emphasis upon patient choice built into the trial design contributed to participant engagement and retention. Accommodating preferences for consultation at home or in clinic was appreciated as some welcomed the opportunity to get out (I.10,15) whilst others welcomed home consultations (I.4,9,17). However, not every participant kept a diary and home visits presented some scheduling difficulties that required out-of-hours working by dietitians, as one participant observed:

> *"You can't do it all the time because the people are at home at different times of the day, some are in the evening, some late evening, they are not there during the day time."* (I.9)

Being able to speak to female dietitians in their language(s) of choice was valued by female participants (I.4,7,11,13), with participants feeling free to take the approach best suited to them:

> *"It made me – like – relaxed when she was around because I didn't, sometimes I don't understand in English, then I could speak to her in Indian. But mostly I didn't want to speak to her in Indian."* (I.16)

Such preferences were less frequently expressed by men (I.18,19).

Relationships

Some participants decided to participate in the trial as a result of knowing members of the research team. In addition, the relationships that developed between participants and their dietitians over the

course of the trial positively contributed to retention. Whilst participants did not commonly refer to or produce for discussion their written materials, they placed importance on good verbal communication (I.11,16), empathy and compassion (I.7,10,18).

> *"She used to advise me but so compassionately and afterwards I used to think over it that it is for my help ultimately I will be the one who will benefit she is not my relative still she cares so much."* (I.19)

Adherence to the intervention

Adherence to the trial proved a complex issue to investigate as participants were well-informed and reported the correct behaviours even if they were not pursuing these. Adherence involved different understandings of the advocated lifestyle changes and unanticipated motivations such as caring for other family members or a sense of obligation to the dietitian. Non-adherence to the trial was in some case culturally motivated, or due to contextual factors such as climate or lack of time.

Understanding adherence

Participants who felt they had adhered to the trial mostly described changes they had made in relation to frequency of exercise (I.11.12) and more informed food choices (I.4,10,11,17,18,21). Some felt that this had not been a particularly significant change as they had a balanced diet already (I.9,12). Conversely, some were evangelical about the trial and their related achievements. Some had lost significant amounts of weight (e.g. *"half a stone"* (I.11)). Two gentleman described how their lives had fundamentally changed, one having given up high alcohol consumption (I.15), the other having become a runner for charity:

> *"I could have never imagined ...I could not even walk 2 kilometres then I have started doing 5 [km] then 10 [km] then I have done lot of 10 then I thought lets go for the bigger [half marathon]then it took me 2hours and 19 minutes ...So with that time we raised £2,000 - I was in the paper!"* (I.19)

Many participants had difficulty in sustaining lifestyle changes (I.10,16,13,14) describing a process of *"fits and starts"* (I.10) as they struggled consistently to follow the advice given despite known health risks. This was true even subsequent to a diagnosis of diabetes (I.7,16). In contrast, looking after other family members influenced adherence. One participant attributed a weight loss of 8kg in part to different methods of food preparation (e.g. less frying) and their having increased their daily exercise due to the need to look after their son's dog (I.8). Another described how they had followed the intervention because they felt it would be beneficial to the whole family (I.11).

Scheduled consultations themselves impacted on levels of adherence as participants either avoided their appointments as they were on the *"downward slope"* (I.10,11) or adapted their behaviour in anticipation:

> *"She used to come in every three months so before she used to come I used to feel a kind of pressure to make sure that my weight had not increased."* (I.18)

Reasons for non-adherence

Culture considerations were evident in discussions regarding the challenges of adherence to the lifestyle intervention. The importance of food within South Asian cultures was referred to for example in the context of sharing home-made sweets with grandchildren (I.2,14) and traditional food preparation techniques:

"Once a week they have children all come so we feel that the food should be much nicer according to the tradition and also children don't like ordinary vegetables they fancy food like from McDonald's so just to compete with that kind of food we try to make our old Indo-Pakistani dishes." (I.20)

The role of food in community functions (I.15) and faith were also given as challenges to adherence, with one participant feeling she couldn't avoid gaining weight:

"I know that if I lose more weight I'm going to put it back on at Ramadan because I just have to walk by food and it just adds onto my body, the fat just is invisible and it runs after you." (I.13)

Whilst the trial was designed to be culturally sensitive to the needs of South Asian populations, some members of the population felt that the advice did not accommodate their more international food preferences (I.10,13,15).

Other reasons for non-adherence related to wider contextual factors. Participants felt they were too busy to find the time to exercise (I.1,4,14,15,16). Two male participants noted the physical demands of their employment as a barrier to exercise (I.14,17), whilst some females felt unable to get enough time out of the house (I.2):

"I try to go for walks but it's hard to find time as I have so many grand-children and women in our community don't get out of the house that much. There is no one else who will come with me." (I.4)

The climate was a consideration for South Asians living in Scotland as participants found recommended exercise such as walking problematic in the cold (I.1,2,15,16) and food was described as a cultural representation of warmth (I.2). It therefore seemed counter-intuitive to increase one without the other.

Discussion

We used qualitative methods to understand the social and behavioural dimensions of the first family-based, culturally adapted randomised lifestyle change trial to investigate approaches to reducing obesity and prevent type II diabetes mellitus in people of Indian- and Pakistani-origin living in Scotland. Findings indicate that individuals may not necessarily prioritise their own health over other factors when making lifestyle choices despite known risks and prevention strategies. Participants were attracted to the trial by the availability of regular health monitoring and personalised information, particularly those who were aware of a family history of diabetes. The choice of home- or clinic-based interventions, communication in the participants' chosen language(s) and trusting relationships between participants and dietitians contributed to the high retention rate. Participants stated that they did not find adherence to advice on food choices and preparation problematic, although some conceded that they did not always choose to take the advice, citing examples of community and faith based considerations that made consistent adherence difficult. Participants did find increasing their levels of physical activity problematic given demands such as the Scottish climate, long working hours, physically demanding jobs and domestic commitments.

One important limitation of this study was the use of interpreters during data generation. We sought to give participants the opportunity to participate in a language of their choosing and could not achieve

For peer review only

this without synchronous translation. During the initial stages of the study retrospective quality checking of interview audio and transcripts highlighted that the interpreter was not accurately reporting the views of the participants. We addressed this by recruiting a new interpreter and providing training as to the exact nature and purpose of the services required. To ensure that the data from the three affected interviews were not lost we had the original audio recording re-translated. We note however that the conduct of these interviews was compromised to a small extent by this limitation. A further limitation is the lack of inclusion of Indian Hindu participants and the small sample of family volunteers. For practical reasons (such as interview cancellation by participants) we could not achieve this within our sample during the time available.

In discussing our findings in the light of current literature on participation and retention we consider the benefits sought from the intervention, flexibility within the trial design and participant/dietitian relationships. Regarding adherence our participants talked about lifestyle changes and we have reported findings accordingly. In contrast, the literature considers attitudes to food and physical activity separately and we discuss our findings in this context.

The need for individualised and context specific information for people with pre-diabetes has been acknowledged[26] and was a benefit participants sought from engaging with the trial. People recognised they were at risk due to family histories. Losing weight was not articulated as a dominant sought-after benefit even though it was a secondary outcome of the trial and this may be due to culturally situated traditional beliefs about body size. In South Asian communities a tendency to equate large size with healthy womanhood, marriage and reproduction, securing a good job and high social status has been identified,[27] although this view may be less prevalent now. Understanding of the causal relationship between weight and diabetes was less evident, re-iterating calls for education within South Asian communities on the causes and prevention of diabetes.[28]

Findings highlight the importance of accommodating participant choice in the design of complex lifestyle interventions. Appreciation of flexibility within diabetes prevention programmes has been recognised,[29] as has the need to overcome known barriers to effectively engaging with minority ethnic communities.[13-15] Whilst culturally sensitive adaptations are essential and were achieved within this trial[16] they should avoid reinforcing stereotypes,[52] for example by focusing on traditional dishes. This did not necessarily sufficiently accommodate complex patterns of dietary acculturation.[31]

Despite cultural adaptation, the trial written resources were rarely referred to. In contrast, verbal communication and participant/dietitian relationships were repeatedly referred to. The importance of non-judgemental professional attitudes, and empathy ~~upon~~ within self-management interventions has been recognised[26,29] and, together with known issues of trust and respect between researchers and marginalised communities, [14,15] was anticipated in the trial design. The intimate nature of this home based lifestyle intervention required a compassionate approach to investigation, out-of-hours working and an emphasis on participant choice different to that of the majority of interventions delivered in clinical settings. Dietitian skill in building relationships was felt to be very important in addition to skills in the verbal communication of advice and information. The importance of continuity in dietician staff was emphasised in trial design, but the strength of participant-dietitian relationships was not anticipated and proved to be significant in retention across the trial (99% in intervention group and 97% in control group). In some cases it seemed that participant loyalty was to the dietitian rather than the trial. This indicates the importance of relational aspects of trial participation that would benefit from further investigation.

For peer review only

Our findings suggest that the cultural construction of food made it difficult for South Asians to make the healthy food choices advised despite known health risks. For members of this community, food is the focus of good living and etiquette.[27] In communities that have previously faced economic insecurity, being able to afford food is a sign of success, demonstrating social power and hospitality.[27,31,32]Although most migrants alter their eating habits following migration,[31] South Asian systemic beliefs regarding the heating and cooling properties and merits of food endure.[33] The role of food in community- and faith-based activities together with complex intergenerational patterns of dietary acculturation detracted from self-reported consistent adherence to both the tailored and low-intensity interventions.

It is known that the South Asian population has lower levels of physical activity than the general population and lesser intrinsic motivations for physical exercise.[34,35] We have corroborated proposed reasons for this, including lack of time and/or childcare[34,35] and gendered barriers to exercise, such as the availability of single-sex facilities and the depiction of exercise as a selfish act detracting from family care.[35] We further note the role of climate and the interplay between South Asian systemic beliefs regarding the heating and cooling properties and merits of food[33]and the demands of living and undertaking physical activity in the cold Scottish climate.

Ourne study considered one research sub-population: the South Asian community living in Scotland. A more general insight from these findings is that well-informed individuals may not necessarily choose or feel able to act on health-related advice due to other lifestyle considerations. People sought health related information as the main benefit of participation and the dietary requirements of the trial were not felt by participants to be an extra burden in their everyday life. However, culturally situated influences on food choices often took precedence over the advice and information given in both intervention and control groups despite acknowledged health implications. This highlights the need for the adaptation of lifestyle interventions to accommodate cultural considerations amongst ethnic minority research participants.

The increased levels of physical activity were however seen to pose an additional burden by participants, again due to cultural factors including those previously identified, such as lower intrinsic motivation, a lack of time and/or access to appropriate facilities, and domestic considerations, each and all of which may impact upon the prevention and management of diabetes In addition, findings highlighted the role of climate in decisions relating to food and physical activity. This suggests that diet and exercise are less distinct within South Asian communities and indicates a need to better understand the impact of climate on lifestyle choices for immigrant populations.

Acknowledgements: We have previously thanked and named many individuals who contributed to the trial.(8) We further acknowledge the Trial Dietitians (Alyson Hutchison, Anu Sharma, Sunita Wallia and Ruby Bhopal), Nandini Choudhuri, Sujama Roy and Salman Aziz; current Trial Steering Committee (Prof Nigel Unwin (ex-chair), Prof Graham Hitman (chair), Dr Nita Forouhi, Dr Deepak Bhatnagar, Dr Ghada Zoubiane , and Mr Iqbal Anwar); the trial Data Monitoring and Ethics Committee (Prof Iain Crombie, Dr Mike Small, Dr Mike Kelly, Prof Kamlesh Khunti); South Asian Community & religious organisations and individuals who contributed to the recruitment efforts; and finally to all the participants who gave their time to take part in this study.

Funding: This study was funded by the National Prevention Research Initiative (G0501310), a funding consortium comprising the British Heart Foundation; Cancer Research UK; Department of

For peer review only

Health; Diabetes UK; Economic and Social Research Council; Medical Research Council; Health & Social Care Research & Development Office for Northern Ireland; Chief Scientist Office, Scottish Government Health Directorate; the Welsh Assembly Government; and World Cancer Research Fund. Additional financial support was provided from NHS Lothian and NHS Greater Glasgow & Clyde R&D, Chief Scientist Office, NHS Health Scotland and NHS National Services Scotland.

Contributors: Zoe Morrison and Aziz Sheikh were responsible for the study design, supervised the data collection, and drafted the manuscript. Anne Douglas and Raj Bhopal designed the protocol for the larger study and assisted with obtaining consent for data collection together with other members of the trial team. Each of the authors was involved in data analysis and interpretation, critical review and refinement of the manuscript.

Competing interests: We declare that we have no conflict of interest.

Data sharing statement: Interview audio recordings and transcripts are available from the corresponding author at the University of Edinburgh, who will provide a permanent, citable and open access home for the dataset.

Formatted: Justified

References

1. Fischbacher C. M., Bhopal R., Steiner M., Morris A. D., Chalmers J. Is there equity of service delivery and intermediate outcomes in South Asians with type 2 diabetes? Analysis of DARTS database and summary of UK publications. *Journal of Public Health*, 2009; 31: 2; 239 – 249. doi 10.1093/pubmed/fdp003.
2. Worth A, Irshad T, Bhopal R, Brown D, Lawton J, Grant E, Murray S, Kendall M, Adam J, Gardee R, Sheikh A. Vulnerability and access to care for South Asian Sikh and Muslim patients with life limiting illness in Scotland: prospective longitudinal qualitative study. *British Medical Journal,* 2009; 338: b183. doi: 10.1136/bmj.b183.
3. Li G, Zhang P, Wang J, Gregg EW, Yang W, Gong Q, et al. The long-term effect of lifestyle interventions to prevent diabetes in the China Da Qing Diabetes Prevention Study: a 20-year follow-up study. *Lancet* 2008 May 24;371(9626):1783-9.
4. Tuomilehto J, Lindstrom J, Eriksson JG, Valle TT, Hamalainen H, Ilanne-Parikka P, et al. Prevention of type 2 diabetes mellitus by changes in lifestyle among subjects with impaired glucose tolerance. *N Engl J Med* 2001 May 3;344(18):1343-50.
5. Knowler WC, Barrett-Connor E, Fowler SE, Hamman RF, Lachin JM, Walker EA, et al. Reduction in the incidence of type 2 diabetes with lifestyle intervention or metformin. *New England Journal of Medicine* 2002 Feb 7;346(6):393-403.
6. Gillies CL, Abrams KR, Lambert PC, Cooper NJ, Sutton AJ, Hsu RT, et al. Pharmacological and lifestyle interventions to prevent or delay type 2 diabetes in people with impaired glucose tolerance: systematic review and meta-analysis. *BMJ* 2007 Feb 10;334(7588):299.
7. Hopper I, Billah B, Skiba M, Krum H. Prevention of diabetes and reduction in major cardiovascular events in studies of subjects with prediabetes: meta-analysis of randomised controlled clinical trials. *Eur J Cardiovasc Prev Rehabil* 2011 Dec;18(6):813-23.
8. Douglas A, Bhopal RS, Bhopal R, Forbes JF, Gill JM, McKnight J, Murray G, Sattar N, Sharma A, Wallia S, Wild S, Sheikh A. Design and baseline characteristics of the PODOSA

For peer review only

(Prevention of Diabetes & Obesity in South Asians) trial: a cluster, randomised lifestyle intervention in Indian and Pakistani adults with impaired glycaemia at high risk of developing type 2 diabetes. *BMJ Open*, 2013; Feb 22;3(2). pii: e002226. doi: 10.1136/bmjopen-2012-002226.

9. Griffiths, C, Motlib, J, Azad,A, Ramsay, J, Eldridge, S, Feder,G, Khanam, R, Munni, R, Garrett, M. Randomised controlled trial of a lay-led self-management programme for Bangladeshi patients with chronic disease. *Br J Gen Pract.* 2005 November 1; 55(520): 831–837.
10. Mason S, Hussain-Gambles M, Leese B, Atkin K, Brown J. Representation of South Asian people in randomised clinical trials: analysis of trials' data. *BMJ* 2003; 326: 1244–1245.
11. Sheikh A, Netuveli G, Kai J, Panesar SS. Comparison of reporting of ethnicity in US and European randomised controlled trials. *BMJ* 2004; 329: 87-8.
12. Samsudeen B S, Douglas A, Bhopal R. Challenges in recruiting South Asians into prevention trials; health professional and community recruiters' perceptions on the PODOSA trial. *Public Health* 2011; 125:201-209.
13. Hussain-Gambles M. South Asian patients' views and experiences of clinical trial participation. *Family Practice* 2004; 21: 636-42.
14. Stirland L, Halani L, Bhopal, R, et al. Recruitment of South Asians into asthma research: qualitative study of UK and US researchers. *Primary Care Respir J.* 2011 Sep;20(3):282-90. Doi:10.4104/pcrj.2011.00032
15. Harris, Y, Gorelick PB, Samuels P, Bempong, I. Why African Americans may not be participating in clinical trials. *J Natl Med Assoc* 1996: 88: 630-634.
16. Wallia S, Bhopal R.S., Douglas A, Bhopal R, Sharma A, Hutchison A, et al. Culturally adapting the prevention of diabetes and obesity in South Asians (PODOSA) trial. *Health Promot Int* 2013
17. Bhopal RS, Douglas A, Walia S, Fobes JF, Lean M, Gill JMR, et al. Weight change over three-years in South Asians at high risk of diabetes: effects and costs in a cluster-randomised trial of a lifestyle intervention. *Lancet Diab Endocrin* *2014:* 2: 218-27.~~(in press)~~
18. Malterud K. Qualitative Research: standards, challenges and guidelines. *Lancet*, 2001:358; 483-488.
19. The PLOS Medicine Editors. Qualitative research: understanding patients' needs and experiences. *PLoS Med* 4(8): e258.doi:10.1371/journal.pmed.0040258
20. Riessman C K. *Narrative Methods for the Human Sciences,* London: Sage; 2008.
21. Squire C. Experience-centred and culturally orientated approaches to narrative. In: Andrews M, Quire C, and Tamboukou M. (eds.) *Doing Narrative Research,* London: Sage; 2008.
22. Patton MQ. (1990) *Qualitative evaluation and research methods.* 2nd ed. London: Sage Publications.
23. QSR International Pty Ltd. NVivo qualitative data analysis software Version 9. 2008.
24. Barbour, R. *Introducing Qualitative Research.* Sage: London; 2008.
25. Morse J. The significance of saturation. *Qualitative Health Research.* 1995;5(2):147-
26. Maindal et al. Lifting the lid of the "black intervention box" –the systematic development of an action competence programme for people with screen-detected dysglycaemia. *BMC Health Services Research* 2010,10: 114.
27. Bush, HM, Williams, RGA, Lean, MEJ and Anderson, AS. Body image and weight consciousness among South Asian, Italian and general population women in Britain. *Appetite*, 2001, 37:207-215.

For peer review only

28. Rankin, R and Bhopal, R. Understanding of heart disease and diabetes in a South Asian community: cross-sectional study tsting the 'snowball' sample method. *Public Health*, 2001; 253-260.
29. Walker, C, Hernan, A, Reddy, P and Dunbar, JA. "Sustaining modified behaviours learnt in a diabetes prevention program in regional Australia: the role of social context. *BMC Health Services Research* 2012, 12: 460.
30. Liamputtong, P. *Researching the vulnerable*. Sage: London; 2007.
31. Azar, KMJ, Chen, E. Holland AT and Palaniappan, LP. Festival Foods in the Immigrant Diet. *J Immigrant Minority Health*. DOI: 10.1007/s10903-012-9705-4. 2012.
32. Joshi, MS, Lamb, R. New foods for old? The diet of South Asians in the UK. Psychol Dev SOc, 2000;12:83-103.
33. Bradby, H. Health, eating and heart attacks: Glasweigian Punjabi women's thinking about everyday food. In Caplan, P (ed.) *Food, Health and Identity*. London: Routledge.1997.
34. Babkus WS and Thompson JL. Physical Activity among South Asian Women: a systematic, mixed methods review. *International Journal of Behavioral Nutrition and Physical Activity,* 2012, 9: 150.
35. Jepson, J. et al, 2012 Physical activity in South Asians: an in-depth qualitative study to explore motivations and facilitators.

For peer review only

14

Table 1: Participant population by geographical location

	Glasgow		Edinburgh		Total	
Research subjects	132		39		171	
	Male	Female	Male	Female	Male	Female
	58	74	20	19	78	93
Family volunteers	114		10		124	
	Male	Female	Male	Female	Male	Female
	28	86	0	10	28	96

Table 2: Qualitative study participants by geographical location

	Glasgow		Edinburgh		Total	
Research subjects	14		6		20	
	Male	Female	Male	Female	Male	Female
	9	6	4	2	13	7
Family volunteers	4		0		4	
	Male	Female	Male	Female	Male	Female
	1	3	0	0	1	3

For peer review only

Table 3: Qualitative study participants by intervention, ethnicity, language and faith

	Trial participants	Randomised group		Ethnicity*		Preferred Language*					Faith*			Family Volunteers	
		15 intervention	4 intervention	Indian	Pakistan	English	Urdu	Punjabi	More than one language	Use of interpreter during interview	None	Muslim	Sikh	15 intervention	4 intervention
Male	12	7	5	6	6	9	0	0	3	3	1	6	5	0	1
Female	8	2	6	3	5	3	2	1	2	4		4	4	1	2
Total	20	9	11	9	11	12	2	1	5	7	1	11	9	1	3

*Self-defined

For peer review only

16

Table 4: Details of interview participants

Interview no.	Location	Randomised group (1=tailored intervention, 2= low intensity intervention)	Gender (M/F)	Ethnicity I=Indian P=Pakistani	Preferred language for speaking	Number of participants (family volunteer)	Researcher
1	Glasgow	2	M	I	Punjabi	1	ZM (and interpreter)
2	Glasgow	2	F	I	Punjabi	1	NC (and interpreter)
3	Edinburgh	1	M	P	English	1	NC
4	Glasgow	2	F	P	Urdu	1	NC
5	Edinburgh	1	F	P	English	1	NC
6	Glasgow	2	F	I	Punjabi	1	NC (and interpreter)
7	Glasgow	2	F	I	Punjabi	(wife)	NC (and interpreter)
8	Glasgow	1	M	I	English	1	NC
9	Edinburgh	2	M	P	English	1	NC
10	Glasgow	1	M	I	English	1	NC
11	Glasgow	2	F	P	Urdu, Hindi, Punjabi	1	NC
12	Glasgow	2	M	I	English	1	NC
13	Glasgow	2	F	P	English	1	NC
14	Edinburgh	1	M	P	English	1	ZM
15	Edinburgh	1	M	I	English	1	ZM
16	Edinburgh	1	F	I	English	1	ZM
17	Glasgow	1	M	P	English	1	ZM
18	Glasgow	1	M	P	Urdu, Punjabi	2 (wife)	ZM (and interpreter)
19	Glasgow	2	M	I	English	2 (brother)	ZM
20	Glasgow	2	M	P	Urdu	1	ZM (and interpreter)
21	Glasgow	2	M	P	English, Urdu	2 (wife)	ZM (and interpreter)

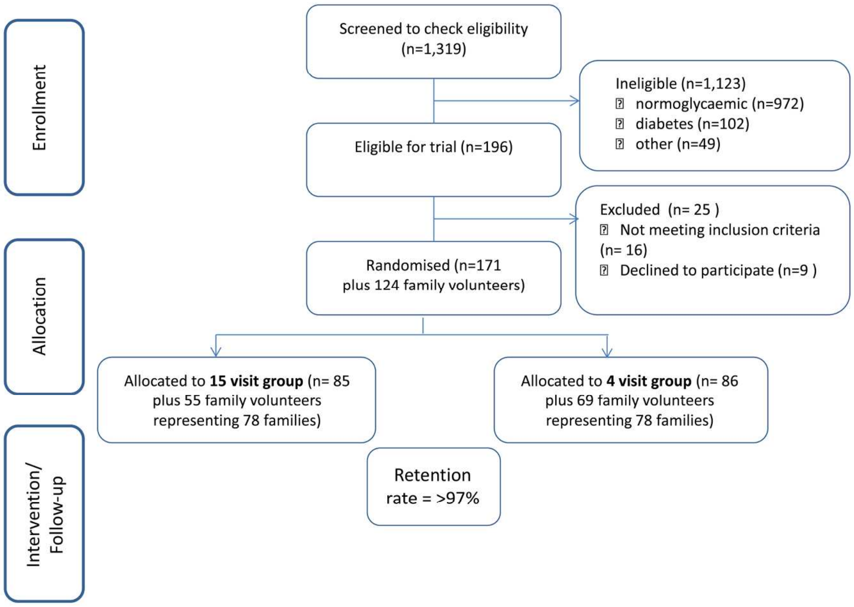

Main trial summary consort flowchart
190x142mm (300 x 300 DPI)

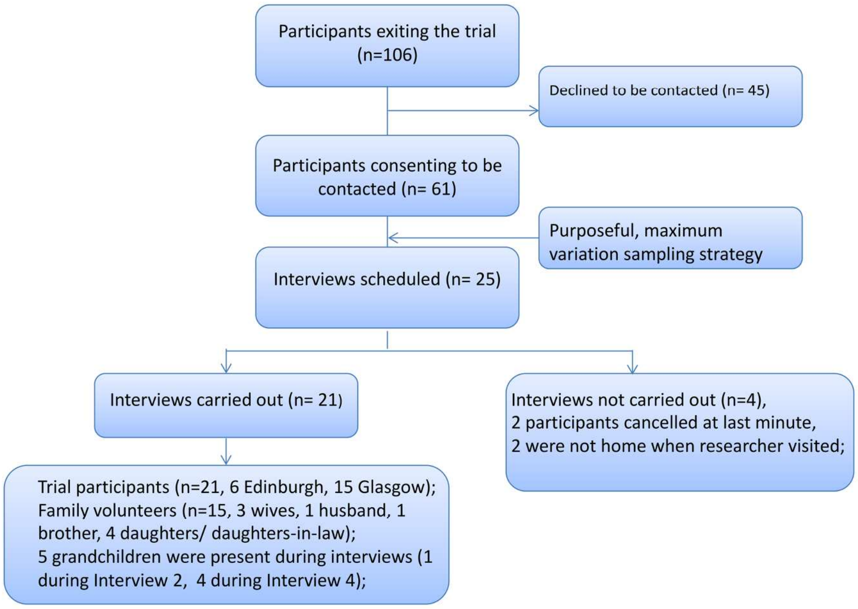

Qualitative study recruitment and data generation
190x142mm (300 x 300 DPI)

Understanding experiences of participating in a weight loss lifestyle intervention trial: qualitative evaluation of South Asians at high risk of diabetes

STROBE 2007 (v4) Statement—Checklist of items that should be included in reports of *cross-sectional studies*

Section/Topic	Item #	Recommendation	Reported on page #
**Title and abstract**	1	(*a*) Indicate the study's design with a commonly used term in the title or the abstract	1,2
		(*b*) Provide in the abstract an informative and balanced summary of what was done and what was found	2
**Introduction**			
Background/rationale	2	Explain the scientific background and rationale for the investigation being reported	3,4
Objectives	3	State specific objectives, including any prespecified hypotheses	3
**Methods**			
Study design	4	Present key elements of study design early in the paper	3,4
Setting	5	Describe the setting, locations, and relevant dates, including periods of recruitment, exposure, follow-up, and data collection	3-4
Participants	6	(*a*) Give the eligibility criteria, and the sources and methods of selection of participants	3-4
Variables	7	Clearly define all outcomes, exposures, predictors, potential confounders, and effect modifiers. Give diagnostic criteria, if applicable	4-7
Data sources/ measurement	8*	For each variable of interest, give sources of data and details of methods of assessment (measurement). Describe comparability of assessment methods if there is more than one group	4-7, 12-16
Bias	9	Describe any efforts to address potential sources of bias	4, 13-15
Study size	10	Explain how the study size was arrived at	4-5
Quantitative variables	11	Explain how quantitative variables were handled in the analyses. If applicable, describe which groupings were chosen and why	N/A
Statistical methods	12	(*a*) Describe all statistical methods, including those used to control for confounding	N/A
		(*b*) Describe any methods used to examine subgroups and interactions	
		(*c*) Explain how missing data were addressed	
		(*d*) If applicable, describe analytical methods taking account of sampling strategy	

For peer review only - http://bmjopen.bmj.com/site/about/guidelines.xhtml

Understanding experiences of participating in a weight loss lifestyle intervention trial: qualitative evaluation of South Asians at high risk of diabetes

		(*e*) Describe any sensitivity analyses	
**Results**			
Participants	13*	(a) Report numbers of individuals at each stage of study—eg numbers potentially eligible, examined for eligibility, confirmed eligible, included in the study, completing follow-up, and analysed	2,3-4, 12-17
		(b) Give reasons for non-participation at each stage	3-4
		(c) Consider use of a flow diagram	
Descriptive data	14*	(a) Give characteristics of study participants (eg demographic, clinical, social) and information on exposures and potential confounders	2,3-4, 12-17
		(b) Indicate number of participants with missing data for each variable of interest	2,3-4, 12-17
Outcome data	15*	Report numbers of outcome events or summary measures	2,3, 13
Main results	16	(*a*) Give unadjusted estimates and, if applicable, confounder-adjusted estimates and their precision (eg, 95% confidence interval). Make clear which confounders were adjusted for and why they were included	N/A
		(*b*) Report category boundaries when continuous variables were categorized	
		(*c*) If relevant, consider translating estimates of relative risk into absolute risk for a meaningful time period	
Other analyses	17	Report other analyses done—eg analyses of subgroups and interactions, and sensitivity analyses	4-7
**Discussion**			
Key results	18	Summarise key results with reference to study objectives	5-7
Limitations	19	Discuss limitations of the study, taking into account sources of potential bias or imprecision. Discuss both direction and magnitude of any potential bias	7
Interpretation	20	Give a cautious overall interpretation of results considering objectives, limitations, multiplicity of analyses, results from similar studies, and other relevant evidence	7-9
Generalisability	21	Discuss the generalisability (external validity) of the study results	7-9
**Other information**			
Funding	22	Give the source of funding and the role of the funders for the present study and, if applicable, for the original study on which the present article is based	9

*Give information separately for cases and controls in case-control studies and, if applicable, for exposed and unexposed groups in cohort and cross-sectional studies.

For peer review only - http://bmjopen.bmj.com/site/about/guidelines.xhtml

Understanding experiences of participating in a weight loss lifestyle intervention trial: qualitative evaluation of South Asians at high risk of diabetes

Note: An Explanation and Elaboration article discusses each checklist item and gives methodological background and published examples of transparent reporting. The STROBE checklist is best used in conjunction with this article (freely available on the Web sites of PLoS Medicine at http://www.plosmedicine.org/, Annals of Internal Medicine at http://www.annals.org/, and Epidemiology at http://www.epidem.com/). Information on the STROBE Initiative is available at www.strobe-statement.org.

For peer review only

For peer review only - http://bmjopen.bmj.com/site/about/guidelines.xhtml
